# Genome-wide cis-expression Quantitative Trait Loci (eQTL) and transcriptomic signals reveal distinct molecular regulation across correlated feed efficiency traits

**DOI:** 10.3389/fgene.2026.1871055

**Published:** 2026-08-21

**Authors:** Steffimol Rose Chacko Kaitholil, Mark H. Mooney, Omar Cristobal-Carballo, Sevda Hosseinzadeh, Faisal I. Rezwan, Aurélie Aubry, Masoud Shirali

**Affiliations:** 1 Institute for Global Food Security, School of Biological Sciences, Queen's University, Belfast, United Kingdom; 2 Sustainable Livestock Systems Branch, Agri-Food and Biosciences Institute (AFBI), Hillsborough, United Kingdom; 3 Department of Animal Science, Faculty of Agriculture, University of Tabriz, Tabriz, Iran; 4 Department of Computer Science, Aberystwyth University, Aberystwyth, Wales, United Kingdom

**Keywords:** average daily gain (ADG), dry matter intake (DMI), feed efficiency (FE), lambs, multi-trait analyses, residual feed intake (RFI), sustainability

## Abstract

**Introduction:**

Feed efficiency (FE) is a complex trait which determines livestock production profitability, yet the molecular mechanisms behind it remain unclear. This study investigated the blood transcriptomic profile of lambs, alongside genotype data with the aim to uncover the genetic basis of FE traits such as absolute dry matter intake (DMI_absolute_), DMI adjusted for body size (DMI_adjusted_), average daily live weight gain (ADG), and residual feed intake (RFI).

**Materials and Methods:**

Bulk RNA-Seq and genotype data were analysed using three complementary approaches: differential gene expression (DGE) analysis, weighted gene co-expression network analysis (WGCNA), and cis-expression Quantitative Trait Loci (cis-eQTL) mapping. These methods were used independently to identify genes and regulatory networks associated with FE traits and to investigate evidence supporting multi-trait candidate gene selection.

**Results:**

DGE analysis revealed 2, 24, 85 and 4 differentially expressed genes for DMI_absolute_, DMI_adjusted_, ADG, and RFI (*P_adjusted_* < 0.05), functionally enriched in sensory perception, ATP-dependent chromatin remodeling, Notch signaling and immune response pathways. 9 gene modules significantly associated with the FE traits (*P* ≤ 0.05) with correlations ranging from *r* = -0.56 to 0.49, were identified using WGCNA. Single nucleotide polymorphism (SNP)-level cis-eQTL analysis identified 93 eSNPs associated with 74 genes (false discovery rate (FDR) < 0.05), while permutation-derived gene level analysis identified 280 eGenes (FDR < 0.2, empirical *P* < 0.03). Across the three analyses, applying thresholds of DGE (*P_adjusted_* < 0.05), WGCNA (correlation, *P* ≤ 0.05), and cis-eQTL gene-level significance (empirical *P* < 0.05), multiple overlapping genes were identified including *DNMT3A, KANSL1, NCOR1* for DMI_adjusted_, *ACOX2, FANCF, CIMIP2B, LOC101115106, ARMH2, LOC132657496* for ADG, and *LOC114114576* for RFI representing regulators of variations in FE.

**Discussion:**

The integration of DGE, WGCNA, and cis-eQTL analyses identified key genes and regulatory mechanisms associated with variation in FE traits. These results highlight that integrated multi-trait candidate gene identification approaches can reveal key genes that lower feed intake while maintaining animal growth, supporting breeding strategies aimed at improving efficiency and long-term economic sustainability in sheep.

## Introduction

Feed efficiency (FE) plays an important role in sustainable animal farming, influencing both profitability and environmental footprint (Dumont et al., 2018; Ellison et al., 2022) and is influenced by diet, growth stage, management system and genetics (Atti and Mahouachi, 2011; Arthy et al., 2018; Santos et al., 2018; Chacko Kaitholil et al., 2024). FE traits such as dry matter intake (DMI_absolute_), DMI relative to animal body size (DMI_adjusted_), average daily live weight gain (ADG), and residual feed intake (RFI) reflect different ways in which an animal utilises food. DMI_absolute_ reflects total demand for feed, influencing supply of nutrients, ruminal function and energy use, whereas DMI_adjusted_ separates feed intake from metabolic use. In practice, DMI_absolute_ is important for managing flocks and budgeting of feed, as it helps in controlling costs and requirement of resources in production systems. In contrast, DMI_adjusted_ enables making meaningful comparisons between heavier and lighter animals, identifying animals that consume more or less feed relative to their body size or weight, helping breeders select animals that are truly feed efficient as opposed to those just larger in size. ADG measures the growth rate of an animal, whilst RFI combines information on feed intake and weight gain to quantify FE regardless of body size (Koch et al., 1963). A combined understanding of these four traits could provide a multi-dimensional approach for assessing FE, but such multi-trait assessments is an area which remains underexplored in livestock, particularly in sheep. Enhancing upon these traits through the use of genetic selection can lower animal production costs while also reducing greenhouse gas emissions such as methane, supporting more sustainable farming.

Genomic selection for FE is a viable alternative to traditional labour-intensive phenotyping methods (Jiang et al., 2024), as it is moderately heritable, with sheep heritability estimates for DMI, ADG, and RFI ranging from 0.25 to 0.49 (Sepulveda et al., 2022), 0.18 to 0.50 (Gowane et al., 2015; Sepulveda et al., 2022), and 0.31 to 0.54 (Tortereau et al., 2020; Zhao et al., 2023b; Chacko Kaitholil et al., 2024) respectively. Genetic correlation between FE traits also shows selection opportunities as DMI is positively correlated with ADG (*R* ^*2*^ = 0.41) (Valente et al., 2025) and RFI (*R* ^*2*^ = 0.619) (Schaub and Posbergh, 2025), while reported correlations between ADG and RFI are almost zero (Cammack et al., 2005). This suggests that selecting animals for lower DMI could lead to reductions in RFI, however, selecting animals based on RFI may not influence ADG, pointing that future breeding programmes should aim for improved FE without compromising other productivity measures.

Although several studies have reported single nucleotide polymorphisms (SNPs) on genes (through genome-wide association studies (GWAS)) associated with both ADG and RFI including *CAST* and *MSTN* (Liu et al., 2023; Zhao et al., 2023a; Zhao et al., 2024; Chacko Kaitholil et al., 2024; Zhang et al., 2025) that have also been implicated in carcass and meat quality traits (Chacko Kaitholil et al., 2024), there exists a significant gap in such studies regarding DMI and in understanding the functional basis of variations in overall FE. The overlap of gene associations with multiple traits (for instance ADG and RFI) underscores the potential of the genetic improvement of FE to improve multiple production-related traits. However, there are limitations to GWAS especially in finding the precise genes and their functions associated with SNPs. Functional genomic approaches including RNA sequencing (RNA-Seq) and weighted gene co-expression network analysis (WGCNA) can help in this regard by providing insights into how genes and pathways control animal feed intake and weight gain. Differential gene expression (DGE) analysis of RNA-Seq data determines key genes whose expression levels directly associate with variations in studied traits, while WGCNA allows the detection of co-expressed gene modules that reflect interconnected biological mechanisms linked to them. Cis-expression Quantitative Trait Loci (cis-eQTLs) are genetic variations acting on local genes rather than distant chromosomes or genes (Shan et al., 2019). Cis-eQTL mapping offers a further complementary approach to identify expression-related SNPs (eSNPs) and associated genes (eGenes) that affect changes in gene activities providing a deeper insight into transcriptional regulation (Gilad et al., 2008). Through identification of links between genetic variants and gene expression, the distinction between gene expression changes due to genomic variations or those from environmental factors can be resolved. While several RNA-Seq studies have focused on RFI (Keel et al., 2018; Zhang et al., 2019), exploration of DMI and ADG-specific gene expression patterns remain less studied in sheep. Addressing this research gap is essential to understand the biological pathways driving growth efficiency to inform more precise breeding.

Peripheral blood constitutes a readily accessible and informative biological matrix for investigating physiological, nutritional, and metabolic processes in livestock (Jégou et al., 2016). Given its minimally invasive nature, ease of collection, and suitability for repeated sampling, blood represents a practical alternative for large-scale studies without the need for surgical procedures or animal slaughter. Although most studies on FE have traditionally focused on metabolically active tissues such as liver or rumen (Alexandre et al., 2015; Mukiibi et al., 2018), there is a growing interest in the use of blood as a surrogate tissue. Recent studies have demonstrated significant associations between whole blood and FE using transcriptomic methods by capturing integrated signals related to immune function, energy metabolism, and stress responses (Messad et al., 2021; Taiwo et al., 2022; Lindholm-Perry et al., 2025). Gene expression profiles and eQTLs derived from whole blood, predominantly composed of white blood cells, can also reflect variations in metabolic activity, immune function, and energy utilisation associated with FE (Dorji et al., 2021). As immune responses lead to energetic costs and influence partitioning of energy towards various cellular processes, transcriptomic variations in immune-related pathways and white blood cell populations may provide further insight into FE variations (Richardson et al., 2002; Rincón Delgano et al., 2011; Lindholm-Perry et al., 2025). Consequently, blood represents a valuable and practical proxy for understanding the molecular mechanisms underlying FE in livestock including sheep. This current study therefore aims to perform a blood-based multi-layer analytical approach including DGE of RNA-Seq data, WGCNA, and cis-eQTL mapping to identify genes and their expression patterns that can be associated with divergent DMI (both absolute and adjusted), ADG, and RFI, and which can be used to genetically select lambs with potential to grow faster while requiring less feed.

## Materials and methods

### Animals and sampling

Samples used in the present study were obtained from a previously conducted trial at the Agri-Food and Biosciences Institute (AFBI), Hillsborough, Northern Ireland, (United Kingdom) (License number: PPL 2915) designed to investigate the effects of microalgae oil (*Schizochytrium* sp.) supplementation on methane production and rumen fermentation. Briefly, thirty-six (Table 1) male cross-bred lambs (Texel x mule (Bluefaced Leicester x Scottish Blackface), average age of 27 weeks, average initial body weight of 34.1 ± 3.0 kg (mean ± standard deviation (SD)) were randomly allocated to four groups with baseline initial bodyweights not differing significantly between groups (ANOVA, *P* = 0.25, Table 1). Lambs were fed a total mixed ration (TMR), offered once daily between 09:00 and 10:00 h, comprising grass silage and concentrate in equal proportions and had *ad libitum* access to water over the experimental period. Diets were formulated to be isoenergetic and isonitrogenous, providing approximately 11.72 MJ/kg dry matter (DM) of metabolizable energy and 13.66 g N/kg DM of metabolizable protein, sufficient to support an anticipated live weight gain of 0.20 kg/day. Dietary groups differed only in the level of microalgae oil inclusion in the concentrate, set at 0.00% (Control, *n* = 9), 0.54% (Low, *n* = 9), 1.08% (Medium, *n* = 9), and 1.62% (High, *n* = 9) on a DM basis. To maintain a consistent total lipid content across treatments, soya oil was incorporated inversely proportional to microalgae oil levels: 0.00% (High, *n* = 9), 0.54% (Medium, *n* = 9), 1.08% (Low, *n* = 9), and 1.62% (Control, *n* = 9). The experimental trial ran for 18 weeks and after 4-week adaptation to dietary changes, feed intakes and live weights were measured in a daily and weekly basis, and body weights were measured on a weekly basis for 14 weeks. The following phenotypic parameters were individually calculated:

$${DMI}_{absolute}=\text{As}-\text{fed feed amount }\left(\text{kg}\right)\times \text{DM}\%$$ (1)

$${DMI}_{adjusted}=\frac{{DMI}_{absolute}\;\left(kg\right)}{Final\;body\;weight\;\left(kg\right)}$$ (2)

$$ADG=\frac{Final\;body\;weight\;\left(kg\right)-initial\;body\;weight\left(kg\right)}{Days\;on\;feed\;}$$ (3)

$$RFI={DMI}_{actual}-{DMI}_{predicted}$$ (4)

where ${DMI}_{predicted}$ was calculated using the equation of Yang et al. (2020). Animals with low RFI exhibit better FE while those with high RFI are considered less efficient (Zhang et al., 2017).

**TABLE 1:** Effects of graded levels of microalgae (*Schizochytrium* sp.) oil supplementation on growth performance and FE traits in lambs. Study lambs were fed varying levels (0.00% (*n* = 9), 0.54% (*n* = 9), 1.08% (*n* = 9), and 1.62% (*n* = 9) of microalgae oil (*Schizochytrium* sp.) on dry matter basis during the experimental period. SEM represents standard error of mean.

Phenotype	0.00% (control) (mean ± sem)	0.54% (low) (mean ± sem)	1.08% (medium) (mean ± sem)	1.62% (high) (mean ± sem)	*P*
Initial Body weight	36.05 ± 1.686	33.72 ± 1.207	34.77 ± 1.027	32.55 ± 0.951	0.25
Final Body weight	50.72 ± 2.500	47.83 ± 2.034	47.11 ± 1.619	45.00 ± 1.483	0.24
DMI_absolute_	1.11 ± 0.060	1.08 ± 0.057	1.03 ± 0.060	0.93 ± 0.039	0.11
DMI_adjusted_	0.022 ± 0.0003	0.022 ± 0.0006	0.021 ± 0.001	0.020 ± 0.0006	0.31
ADG	0.19 ± 0.013	0.18 ± 0.014	0.16 ± 0.013	0.16 ± 0.019	0.41
RFI	0.02 ± 0.017	0.06 ± 0.036	0.05 ± 0.049	−0.004 ± 0.034	0.54

At the end of the trial period, peripheral blood was collected in the mornings, 3 h after fasting using PAXgene Blood RNA tubes (BD, Franklin Lakes, New Jersey, United States; and QIAGEN, Venlo, Netherlands). All animal study work was conducted at AFBI Hillsborough, under the regulations of the Department of Health for Northern Ireland, in accordance with the requirements of the United Kingdom Animals (Scientific Procedures) Act 1986 and with the approval of the AFBI (Hillsborough) Animal Welfare and Ethical Review Body.

### RNA extraction, sequencing and quality control of RNA-Seq data

Total RNA was isolated from the acquired whole blood using PAXgene Blood RNA Kits (Qiagen) following manufacturer’s protocols which then underwent library preparation using TruSeq3 RNA Library Prep Kit (Illumina, San Diego, California, United States) and sequencing in a commercial lab using the NovaSeq (Illumina, San Diego, California, United States) platform, generating paired-end 150bp reads at a read depth of ∼30 million reads per sample. To ensure quality, the sequenced data generated underwent sample quality control (QC) using FastQC (v0.12.1) (Andrews, 2010) and reads with Phred quality scores below 30 were removed. Subsequently, raw reads underwent pre-processing, including trimming of the adapter sequences and filtering for poor quality reads with Trimmomatic (v0.39) (Bolger et al., 2014) software using the following parameters: ILLUMINACLIP:TruSeq3-PE.fa:2:30:10:2, LEADING:3, TRAILING:3, and MINLEN:36. Trimmed reads were then aligned to the sheep (*Ovis aries*) reference genome [Genome assembly, ARS-UI_Ramb_v3.0 (NCBI, 2023)] available in the National Centre for Biotechnology Information (NCBI) using the HISAT2 (v2.1.0) (Kim et al., 2019) software applying default settings with end-to-end alignment mode. A count matrix, representing the number of mapped reads for each gene, was generated using featureCounts (v2.0.1) (Liao et al., 2014) and the following Parameters: -t exon, and -g gene_id, using the NCBI GTF annotation file. To filter out genes with low expression (Sha et al., 2015), a stringent filtering criterion was applied where genes with zero counts and those less than five counts in more than 70% samples were excluded. Effects of varying levels of microalgae oil inclusion on the analysed FE traits were evaluated using ANOVA. Additionally, outlier samples were identified using a standardized connectivity approach (Z.k), where samples with Z.k values less than −2 were excluded (Fu et al., 2019). Principal component analysis (PCA) was used to assess any sources of confounding variation including population structure and clustering by dietary microalgae oil intake. The first two principal components (PC1 and PC2) were examined, and samples were evaluated for clustering with respect to varying levels of microalgae oil.

### Identification of differentially expressed genes and pathways

DGE analyses were conducted using DESeq2 (Love et al., 2014) in R statistical software (v 4.4.2) (Ihaka and Gentleman, 1996). For this, the count matrix was normalized using the median-of-ratios method, which accounts for sequence depth variations and sample RNA composition. For each gene, dispersion estimates were calculated followed by fitting negative binomial generalized linear model to the normalized counts. The design formula specified DMI_absolute_, DMI_adjusted_, ADG, and RFI as the main factors of interest, with separate model fitted for each, and were treated as continuous exposures, as the studied phenotypes are measured on a continuous scale and not in discrete categories. This type of continuous analysis does not result in any loss of within-group phenotypic variations as observed in categorical trait analyses (Seo et al., 2016). As a sensitivity analysis, DGE analysis was repeated with microalgae oil inclusion as an additional covariate in the DESeq2 design to evaluate the robustness of FE-associated transcriptomic signals. Wald tests were used to find the association between gene expression and the four FE traits, and log_2_ fold changes were calculated, along with multiple testing correction to control for false discovery rate (FDR) using the Benjamini–Hochberg (BH) method. A cut-off threshold was established for the adjusted *P*-value (henceforth, *P*_*adjusted*_) from the Wald test and log_2_ fold change, specifically with *P*_*adjusted*_ less than 0.05 and an absolute log_2_ fold change (|log_2_ fold change|) ≥ 2 (4-fold-change) (Gao et al., 2026), to identify significant differentially expressed genes (DEGs). For DMI_adjusted_, the phenotypic distributions were narrow compared to other traits ranging between 0.017 and 0.026 kg DM/day, hence, regression coefficients were interpreted in the context of the observed phenotypic range [β × (0.026–0.017)] to provide biologically meaningful interpretation of effect sizes. This rescaling was applied for interpretation only and did not affect statistical testing. Significant DEGs (*P*_*adjusted*_ < 0.05, |log_2_ fold change| ≥ 2) were then subjected to further downstream analyses including over-representation analysis (ORA) using clusterProfiler (Yu et al., 2012) to extract significant gene ontology (GO) and Kyoto encyclopedia of genes and genomes (KEGG) pathways based on AnnotationHub (v 3.12.0) (Morgan et al., 2019) (hub: AH114633) GO entry for sheep. GO enrichment analysis was performed using the enrichGO function with all ontology categories considered (Biological Process, Molecular Function, and Cellular Component; ont = “ALL”). KEGG pathway analysis was conducted using the enrichKEGG function with the organism code set to *Ovis aries* (“oas”). Statistical significance for GO terms and KEGG pathways were assessed using a hypergeometric test, and multiple testing correction performed using the BH method. Enriched terms were considered significant at *P*_*adjusted*_ (FDR) < 0.05.

### Co-expressed gene module detection

Co-expressed gene evaluation was performed using the WGCNA (Langfelder and Horvath, 2008) R package to identify genes that are co-expressed with the four FE traits using the normalized counts generated from DESeq2 as the input. The “goodSamplesgenes” and “hclust” parameters were used to identify any missing values and outlier samples. A soft threshold power corresponding to a scale-free topology index (*R* ^*2*^) greater than 0.80 was chosen to promote a scale-free network structure followed by calculation of adjacency matrix. The topological overlap matrix (TOM) was then computed from the adjacency matrix, and its corresponding dissimilarity matrix (1-TOM) was generated. For identification and construction of co-expressed gene modules, a dynamic tree cut algorithm was used with cut height of 0.99, minimum gene module size of 50 and DeepSplit of 2. Co-expressed gene modules were assigned arbitrary colors for the ease of reference and similar modules were merged using the mergeCloseModules function using a cutHeight of 0.25. To summarize gene expression variation within each module, module eigengenes were computed as the first principal component which were calculated using the “moduleEigengenes” option. Correlations between module eigengenes and the four FE traits were assessed using the Pearson correlation method to identify trait-associated modules (*P* ≤ 0.05). Functional enrichment analyses were carried out on significant trait-associated modules (*P* ≤ 0.05) using clusterProfiler R package applying the same parameter settings as in the differentially expressed genes’ pathway analysis to retrieve KEGG pathways.

### Identification of genetic variants regulating gene expression

28 out of the 36 animals were genotyped and genotype data was generated using the OvineSNP50 BeadChip which was then merged using PLINK v1.9 (Purcell et al., 2007) using the merge-list command. Following merging, a QC procedure was applied, consisting of removal of individuals with more than 10% missing genotypes, exclusion of SNPs with more than 10% missing data, removal of SNPs with minor allele frequency (MAF) < 0.01 and those that deviate from the Hardy-Weinberg equilibrium (HWE) less than 10^–6^. In addition, linkage disequilibrium (LD) pruning was conducted using PLINK (--indep-pairwise 50 5 0.8) to reduce redundancy among highly correlated SNPs across the genome. The normalized gene expression matrix from the DGE analysis was loaded, and SNP-level cis-eQTL mapping was performed in R using the MatrixeQTL package (Shabalin, 2012) under a linear model, considering a window of ±1 Mbp. The application of cis-window of ±1 Mbp was consistent with previous sheep eQTL studies (Yuan et al., 2021; Xie et al., 2026) where variants within 1 Mbp of a gene were considered as potential cis-regulators. This window size also reflects LD patterns in sheep, where LD decays rapidly with distance, supporting the use of a broader interval to capture proximal regulatory variation (Mucha et al., 2015). LD-based clumping was performed using PLINK with an *r* ^2^ threshold of 0.8 and a 1 Mb window to identify independent association signals. The 1 Mb window was used for LD clumping to match the cis-eQTL definition and to account for the relatively low SNP density of the OvineSNP50 array, ensuring adequate capture of LD structure. A gene level permutation-based method was then employed to account for SNP density, where genotypes were kept fixed while expression values were permuted across 28 samples with matched genotype and RNA-Seq data followed by repeating cis-eQTL mapping for each permutation. The minimum cis-eQTL *P*-value (*P*) for each gene across all SNPs in the analysed cis window were recorded. This step was repeated for 1,000 permutations and a null distribution of minimum *P* were generated for each gene. An empirical *P* which represents the probability of observation of cis-eQTL signal under the null hypothesis of no genetic regulation, was then calculated for each gene by comparing *P* from the observed analysis to the corresponding null distribution derived from permutation. The resulting empirical *P-values* were then adjusted using the BH FDR method to control for multiple testing. Significant SNP-level cis-eQTL results were merged with SNP annotation data and visualized as a Manhattan plot using the qqman package (Turner, 2018). All preprocessing and analytical steps were conducted on an Ubuntu Linux platform (22.04.5 L TS) using R version 4.4.2.

## Results

### Phenotypic variations within FE traits

Dietary inclusion of microalgae oil did not result in significant changes in the analysed FE traits, with the PCA plot showing no clear separation among treatment groups (Supplementary Figure S1). Consistent with this, statistical comparisons using ANOVA revealed no significant differences across the four dietary treatments (Table 1; Supplementary Figure S2) (*P* > 0.05). Initial and final body weights of lambs were comparable across the four different dietary groups (*P* = 0.25 and *P* = 0.24, respectively). Similarly, no significant differences were observed in DMI_absolute_ (*P* = 0.11) or DMI_adjusted_ (*P* = 0.31). ADG and RFI also remained unaffected by increasing levels of microalgae oil (*P* = 0.41 and *P* = 0.54, respectively). Although a numerical decline in overall feed intake and growth rate was observed with increasing microalgae oil inclusion, these differences were not statistically significant.

### RNA-seq data generation, alignment, and quality assessment

RNA sequencing generated a dataset comprising 2,073,665,072 paired end reads, each with 150bp length, across 36 samples. The sequencing depth per sample varied, with individual sample read counts ranging from 24.2 to 35.1 million. Initial sample pre-processing resulted in the exclusion of two samples (7073, 7085, Supplementary Table S1) due to the presence of many duplicated sequences. Alignment of the sequenced reads to the reference genome of sheep (*O. aries*, ARS-UI_Ramb_v3.0, NCBI, GCF_016,772,045.2) revealed high mapping efficiency, ranging from 81.7% to 94.8% of the total reads aligning uniquely to the genome. Subsequent gene-level quantification revealed that 63.4% to 77.5% of the aligned reads across all the remaining samples were successfully mapped to the annotated gene features in the ARS-UI_Ramb_v3.0 genome assembly, enabling the detection of 31,304 genes. One additional sample (7072, Supplementary Table S1) was removed at this stage due to fewer than 60% of reads being assigned to gene features. Three samples (7094, 6977 and 6764) had connectivity (Z.k) values less than −2 which were considered as outliers and hence discarded (Supplementary Table S1). Consequently, further downstream analyses were carried out on the remaining 30 samples. To ensure robust downstream analysis, genes with low variances were excluded, resulting in the removal of 11,532 genes. A total of 19,772 high-confidence genes were retained for further DGE, ORA, WGCNA, and cis-eQTL mapping.

### Differentially expressed gene profiles and functional enrichments associated with FE

**FIGURE 1:**
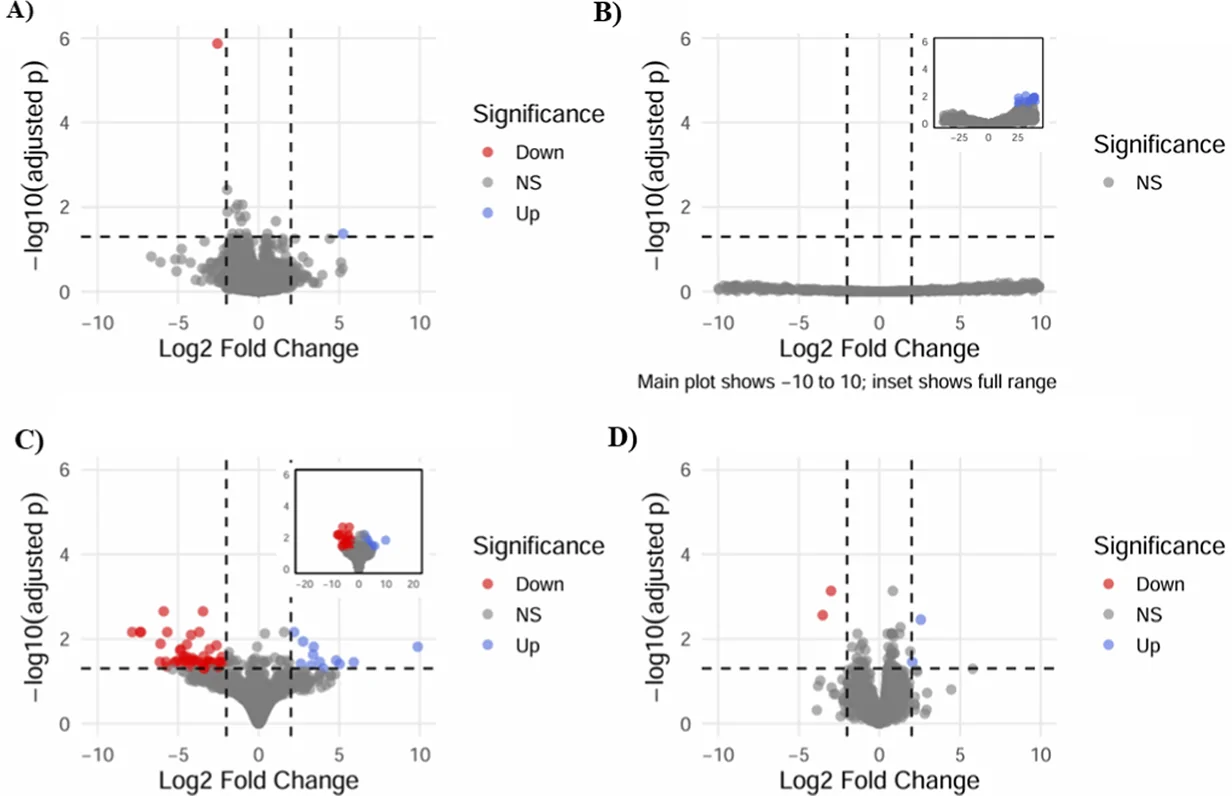
**(A–D)** DEGs associated with DMI_absolute_ **(A)**, DMI_adjusted_ **(B)**, ADG **(C)**, and RFI **(D)**. The plot displays Log_2_ fold change on the x-axis and -log_10_ (P) on the y-axis for all genes tested. Significant DEGs (P_adjusted_ < 0.05, |log2 fold change| ≥ 2) are highlighted, with up/down-regulated genes shown on the left and right as red and blue points. NS represent non-significant genes (grey points). To enhance visual comparability across all traits, symmetric x-axis limits (±10 log_2_ fold change) were applied to all panels. Genes exceeding this range are plotted as an inset panel.

Pre-processed RNA-Seq data were analysed for DGE, treating the four FE traits as continuous exposures in independent models to identify genes differing in transcript abundance across the studied lambs. Since the traits are treated as continuous exposures, the identified DEGs reflect changes in gene expression per unit increase in the studied traits. DEGs were defined based on BH-corrected *P*_*adjusted*_ *< 0.05* combined with |log_2_ fold change| ≥ 2. Only a limited DEGs were identified for DMI_absolute_ with one gene upregulated and another downregulated (Supplementary Table S2; Figure 1A). On the contrary, 24 DEGs were identified for DMI_adjusted_, with all being upregulated. However, scaling the regression coefficients of these 24 DEGs to the observed phenotypic range resulted in only modest expressions (Supplementary Table S2; Figure 1B). For ADG, a total of 85 DEGs were detected (65 upregulated and 20 downregulated) (Supplementary Table S2; Figure 1C), while 4 DEGs were associated with RFI (2 upregulated and 2 downregulated) (Supplementary Table S2; Figure 1D). Notably, no overlapping genes were observed between the four FE traits (Supplementary Figure S3). Sensitivity analysis utilising microalgae oil inclusion as a covariate in the DESeq2 model, revealed several genes across three traits-DMI_absolute_, DMI_adjusted_, and ADG (Supplementary Table S3) overlapping with those obtained from the model generated without microalgae oil in the design. DMI_absolute_-associated DEGs were enriched in sensory, endocrine, immune, and signal transduction pathways, while DMI_adjusted_-associated DEGs were enriched in ATP-dependent chromatin remodelling pathway (Table 2). Three ADG-associated DEGs were functionally enriched in the Notch signalling pathway, and RFI-associated DEG was enriched in immune system, transport, and catabolic pathways (Table 2).

**TABLE 2:** KEGG pathways significantly enriched for DEGs associated with the four FE traits. Significance of the enrichments were assessed using the BH correction method. Those pathways with *P*_*adjusted*_ < 0.05 were considered significantly enriched.

Phenotype	KEGG ID	Pathway name	*P*_*adjusted*_	Genes
DMI_absolute_	oas04740	Olfactory transduction	0.0382	*RGS2*
	oas04670	Leukocyte transendothelial migration	0.0382	*CD99*
	oas04514	Cell adhesion molecule (CAM) interaction	0.0382	*CD99*
	oas04921	Oxytocin signalling pathway	0.0382	*RGS2*
	oas04022	cGMP-PKG signalling pathway	0.0382	*RGS2*
DMI_adjusted_	oas03082	ATP-dependent chromatin remodeling	0.0002	*TRRAP, BAZ1B, ARID1A, EP400, SRCAP*
ADG	oas04330	Notch signaling pathway	0.0134	*APH1B, ATXN1, PSENEN*
RFI	oas04640	Hematopoietic cell lineage	0.0208	*CD24*
	oas04148	Efferocytosis	0.0208	*CD24*

*RGS2*, Regulator of G-protein signalling 2; *CD99*, Cluster of Differentiation 99; *TRRAP,* Transformation/transcription domain associated protein; *BAZ1B,* bromodomain adjacent to zinc finger domain 1B*; AR1D1A,* AT-Rich Interaction Domain 1A*; EP400,* E1A binding protein P400; *SRCAP,* Snf2 related CREBBP, activator protein; *APH1B,* aph-1, homolog B, gamma-secretase subunit; *ATXN1*, ataxin 1; *PSENEN*, presenilin enhancer, gamma-secretase subunit; *CD24*, CD24 molecule.

### FE-associated co-expressed gene modules

**FIGURE 2:**
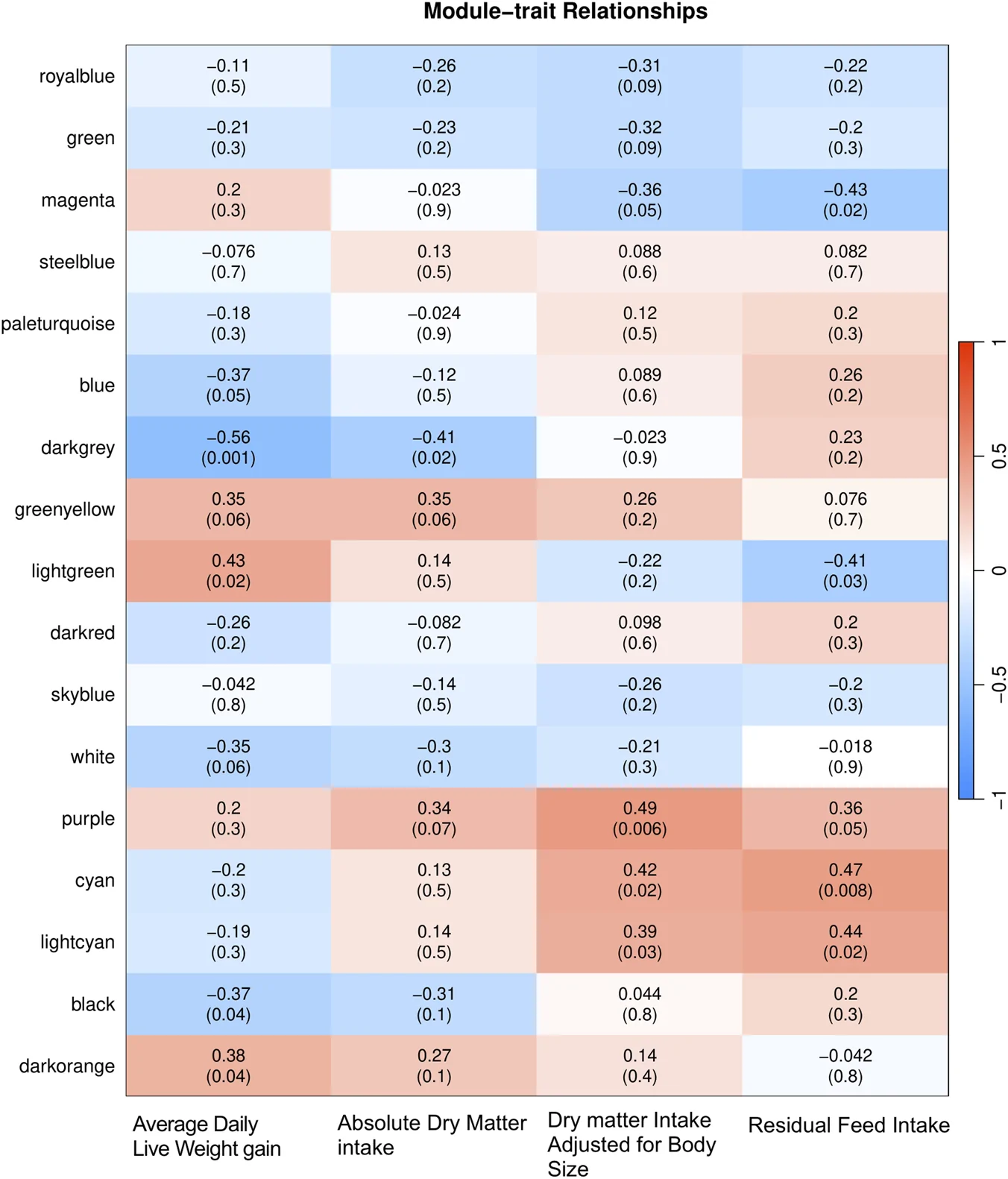
Correlation between WGCNA gene modules (y-axis) and FE traits (x-axis). Each cell contains correlation coefficient between the module and FE trait, along with their *P-*value (in brackets). Modules with *P* ≤ 0.05 were considered as significant.

**FIGURE 3:**
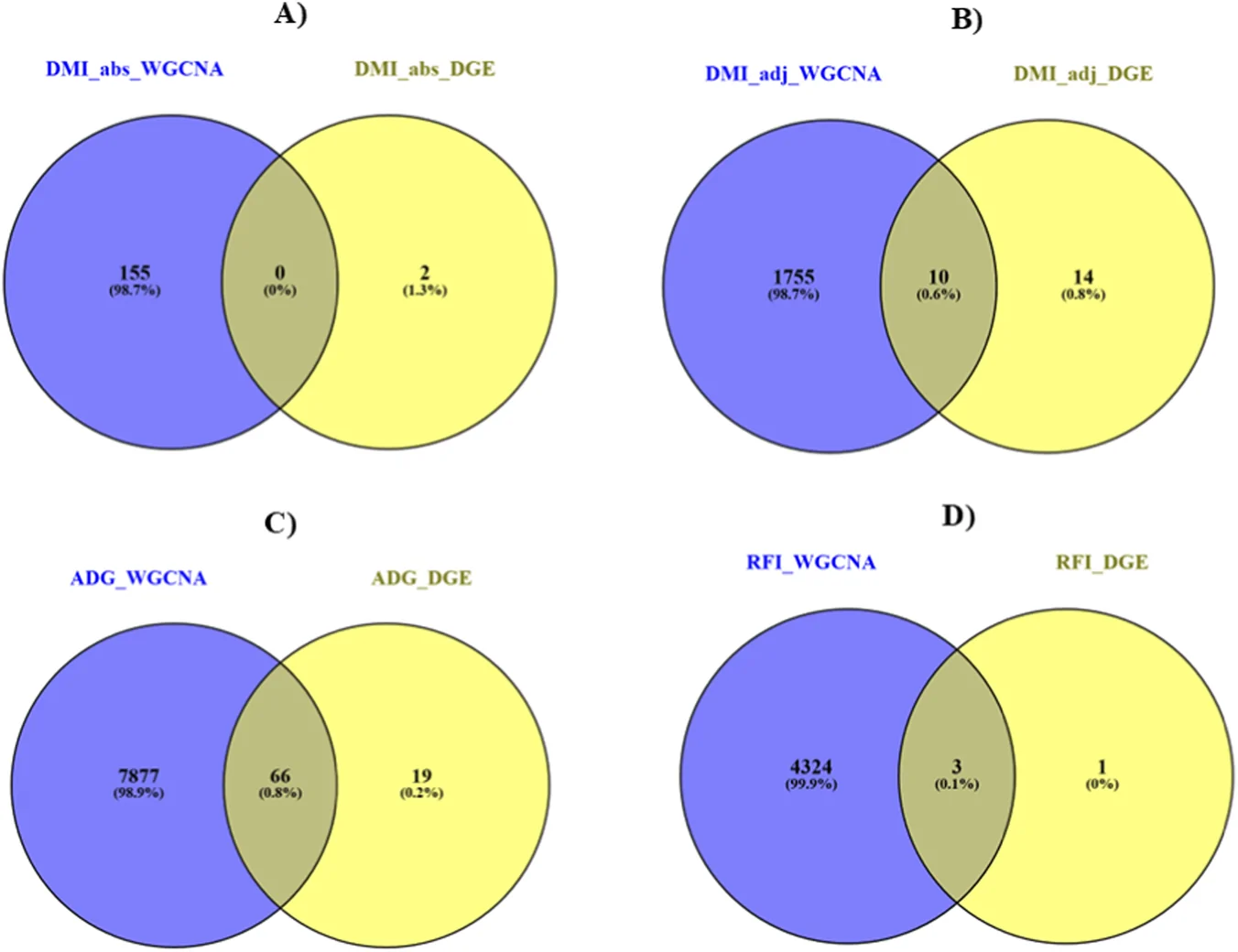
**(A–D)** Overlapping genes identified between WGCNA significant modules and DGE analysis for the studied FE traits. The small set of overlapped genes represents transcriptional signals consistently linked to FE traits in studied lambs.

To complement the gene-level DGE analysis and to uncover the coordinated gene expression patterns underlying FE variations, WGCNA was performed. The soft threshold power was chosen as 4 to construct the network, as it had an *R* ^*2*^ of 0.89, and mean connectivity of 175 (Supplementary Table S4). The dynamic tree cut algorithm divided the input genes (*n* = 19,772) into 17 gene modules. The TOM generated gene clusters are shown in Supplementary Figure S4. Several significant gene modules (*n* = 9, *P* ≤ 0.05) exhibiting correlations with FE traits were identified (Table 3; Figure 2). Notably, the darkgrey module was found to be significantly negatively correlated with DMI_absolute._ In contrast, three modules, namely, lightcyan, cyan, and purple were positively correlated with DMI_adjusted_. The ADG trait was significantly correlated with five modules, namely, darkorange, black, lightgreen, darkgrey, and blue. RFI showed positive correlations with the cyan and lightcyan modules, while negative correlation with the lightgreen and magenta modules. Interestingly, some of the identified significant gene modules were associated with multiple traits. For instance, the cyan and lightcyan modules were associated with DMI_adjusted_ and RFI, darkgrey with DMI_absolute_ and ADG, and lightgreen with RFI and ADG. Notably, 10 genes from DMI_adjusted_-associated modules, 66 genes from ADG-associated modules, and 3 genes from RFI-associated modules overlapped with the DGE analysis identified earlier (Figures 3A–D; Supplementary Table S5).

**TABLE 3:** Gene-co-expression modules significantly correlated with studied FE traits. 9 modules identified through WGCNA are listed alongside their correlations, *P* and direction of correlation for each FE trait.

Module	Significant associations with phenotype	Correlation (*P)*	Direction of correlation
Darkorange	ADG	0.38 (0.04)	Positive
Black	ADG	−0.37 (0.04)	Negative
Lightcyan	DMI_adjusted_	0.39 (0.03)	Positive
Lightcyan	RFI	0.44 (0.02)	Positive
Cyan	DMI_adjusted_	0.42 (0.02)	Positive
Cyan	RFI	0.47 (0.008)	Positive
Purple	DMI_adjusted_	0.49 (0.006)	Positive
Lightgreen	ADG	0.43 (0.02)	Positive
Lightgreen	RFI	−0.42 (0.03)	Negative
Darkgrey	DMI_absolute_	−0.41 (0.02)	Negative
Darkgrey	ADG	−0.56 (0.001)	Negative
Blue	ADG	−0.37 (0.05)	Negative
Magenta	DMI_adjusted_	−0.36 (0.05)	Negative
Magenta	RFI	−0.43 (0.02)	Negative

FE, feed efficiency; DMI, dry matter intake; RFI, residual feed intake.

Functional analysis of genes in the 9 significant co-expressed modules (Figure 2) revealed enrichment for multiple biological processes (Supplementary Table S6), underscoring the functional relevance of the gene sets. Signal transduction, nervous and endocrine system-related pathways were found to be enriched for the modules associated with DMI_absolute_, DMI_adjusted_ and RFI. ADG-associated modules showed enrichments in transport and catabolic pathways, energy metabolism, development, and immune system-related pathways.

### Cis-eQTL mapping reveals regulatory variants with large expression effects

**FIGURE 4:**
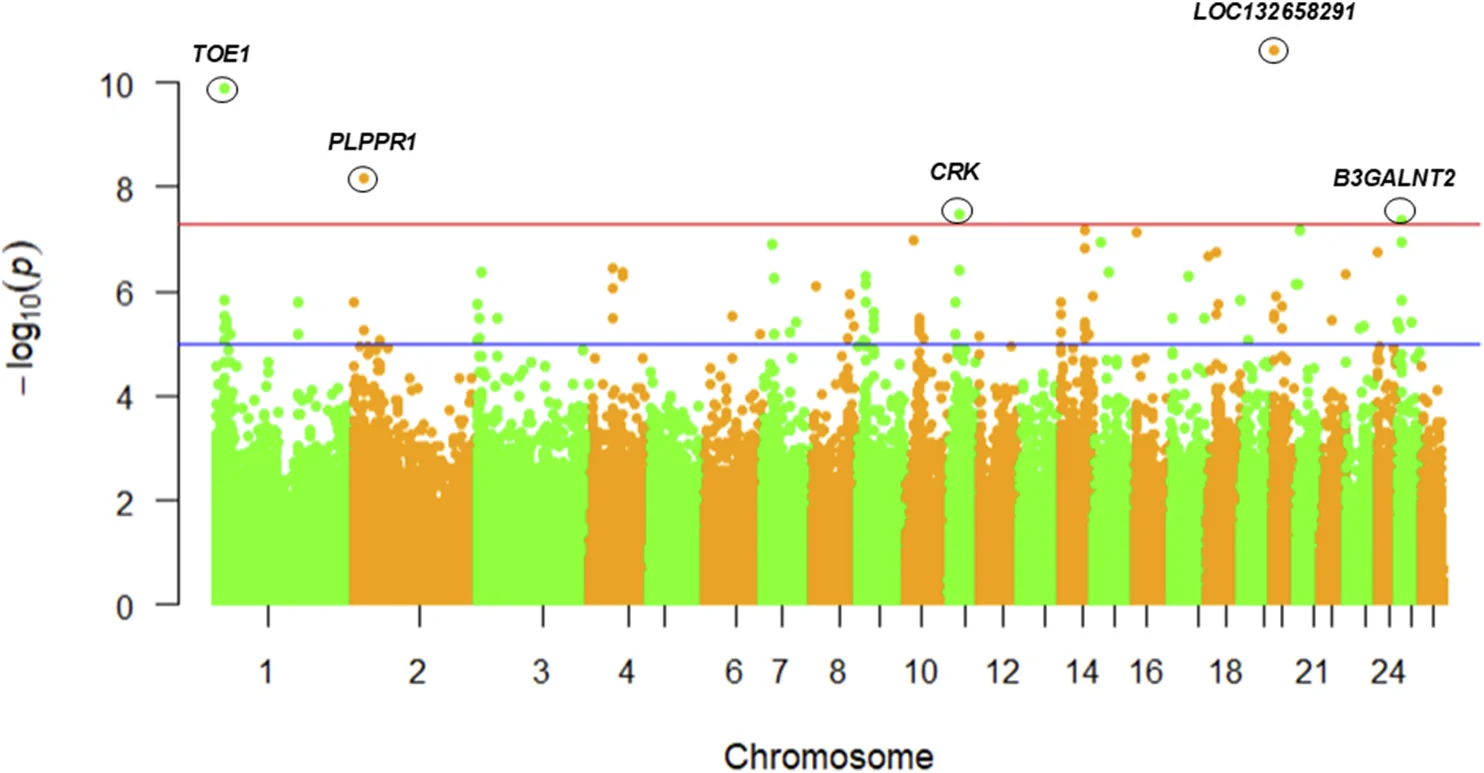
Genome-wide distribution of cis-eQTL associations in studied lambs. Each dot corresponds to SNPs tested for its association with gene expression levels. The x-axis represents all autosomal chromosomes (1–26) and y-axis shows the -log_10_(*P*). Top 5 genes with smallest FDR are labelled representing the most significant SNP-level associations and do not necessarily overlap with genes identified through gene-level permutation analysis. Red horizontal line represents genome-wide significance (-log_10_ (5 × 10^−8^)), while blue horizontal line represents suggestive significance level (-log_10_ (1 × 10^−5^)).

Of the total 51,504 variants, 4,584 were removed due to missing SNP genotypes, 2,175 were excluded due to a MAF <0.01, 738 were removed based on HWE (P < 1 × 10^−6^), and 290 were excluded following LD pruning. This resulted in 43,717 retained variants for subsequent cis-eQTL analysis. All 28 lambs passed QC, with no individuals excluded due to missing genotype data. A total of 603,446 SNP-gene association tests were performed, corresponding to all SNPs located within a ±1 Mbp cis-window of 19,514 genes (in 28 samples). Following LD-based clumping (*r* ^2^ = 0.8, 1 Mbp window), SNP-level multiple testing revealed 93 independent eSNPs, representing 74 unique eGenes (Supplementary Table S7), with the strongest peaks corresponding to genes such as *TOE1* (*P* = 2.66 × 10^−5^, beta = 0.19), *PLPPR1* (*P* = 1.03 × 10^−3^, beta = 0.66), *CRK* (*P* = 4.01 × 10^−3^, beta = −0.29), *LOC132658291* (*P* = 1.44 × 10^−5^, beta = 0.85), and *B3GALNT2* (*P* = 4.46 × 10^−6^, beta = 0.38) (Figure 4). Permutation-based gene-level analysis revealed no genes to surpass the strict FDR cutoff of 0.05, however, 280 eGenes displayed suggestive cis-regulatory effects (FDR, *P* < 0.2, empirical *P* < 0.03), with top signals observed on the same eGenes (*TOE1, PLPPR1, CRK, LOC132658291,* and *B3GALNT2*) identified from the SNP-level cis-eQTL analysis. Interestingly, all 74 genes (Supplementary Table S7) identified from SNP-level analysis were also identified from the gene-level analysis.

### Overlapping genes from integrated DGE, WGCNA, and Cis-eQTL mapping analyses

Overlapping gene sets were identified based on three criteria: (i) FE-associated DEGs with *P*_*adjusted*_ < 0.05 and |log_2_ fold change| ≥ 2 (ii) genes belonging to significant WGCNA modules (correlation, *P* ≤ 0.05), and (iii) permutation-derived cis-eQTL eGenes with empirical *P* < 0.05. The results revealed a small robust set of candidate high-confidence genes associated with the four analysed traits (Table 4). No genes overlapped between the three analyses for DMI_absolute_, while *LOC114114576* overlapped in the three analyses for RFI. Six genes (*ACOX2*, FANCF, *CIMIP2B, LOC101115106, ARMH2, LOC132657496*) appeared common for ADG, while three genes (*DNMT3A, KANSL1, NCOR1*) appeared common for DMI_adjusted_. It should be noted that cis-eQTL significance for overlap analysis was determined at the gene level using permutation-based empirical *P*. Consequently, some overlapping genes reported here may not appear in Supplementary Table S7 which includes only SNP-level associations passing the FDR < 0.05 threshold. Notably, the top SNP-level signals (*TOE1, PLPPR1, CRK, LOC132658291,* and *B3GALNT2*) were not retained in the final overlapping gene sets, as candidate overlapping genes were defined based on gene-level permutation criteria and integration across multiple analytical approaches, rather than SNP-level significance alone. Furthermore, several substantial overlapping genes were observed between WGCNA modules and cis-eQTLs, with the largest number of genes shared among blue module and cis-eQTL (*n* = 43), followed by lightgreen module (*n* = 40), and black (*n* = 31) (Supplementary Table S8).

**TABLE 4:** Candidate overlapping genes identified from the three methods for the studied FE traits. Overlap suggests convergence of evidence from RNA-Seq based DGE (*P*_*adjusted*_ < 0.05), WGCNA (correlation, *P* < 0.05), and cis-eQTL (empirical *P* < 0.05). No overlapping genes were observed for DMI_absolute_.

Gene	Lead SNP	Chromosome, position	Beta	Empirical *P*	FDR	Gene functions
Dry matter intake adjusted for body weight (DMI_adjusted_)						
DNMT3A	s14411.1	3, 33009018	−0.14	0.002	0.2	epigenetic regulation (Chen and Chan, 2014), adipogenesis (Qimuge et al., 2019), and muscle development (Fan et al., 2020)
*KANSL1*	OAR11_46289441.1	11, 46289441	0.16	0.02	0.37	Immune responses, mitosis and cell proliferation (Meunier et al., 2015)
*NCOR1*	OAR11_33214497.1	11, 33214497	−0.05	0.04	0.45	Processing fat and maturation of muscles (Mirzapour et al., 2023)
Average daily live weight gain (ADG)						
*ACOX2*	DU174410_88.1	19, 42472374	−0.23	0.008	0.28	lipid metabolism, regulation of homeostasis (Cao et al., 2025; Cao, 2017)
FANCF	s74518.1	21, 18928075	−0.18	0.008	0.28	DNA repair mechanism (Papadaki et al., 2025)
CIMIP2B	OAR2_53127735.1	2, 53127735	0.35	0.01	0.34	Ciliary and flagellar structure and function (GeneCards, 2025)
*ARMH2*	OAR20_32896798.1	20, 32896798	−0.18	0.03	0.43	Functioning of sperms (Zhao et al., 2025)
*LOC132657496*	OAR12_6103280.1	12, 6103280	−0.58	0.003	0.21	Uncharacterized
*LOC101115106*	OAR3_44467884.1	3, 44467884	−0.36	0.03	0.44	uncharacterized
Residual feed intake (RFI)						
*LOC114114576*	OAR4_113848235.1	4, 113848235	0.29	0.03	0.41	hematopoietic stem cell maintenance, mitochondrial permeability (Zhou et al., 2024)

FE, feed efficiency; DMI, dry matter intake; RFI, residual feed intake.

## Discussion

Transcriptomic studies on FE traits have identified numerous genes and biological pathways associated with variation in this complex trait across livestock species. However, large-scale application of such studies are often constrained by the need for invasive or semi-invasive tissue sampling (Lindholm-Perry et al., 2025) which may limit both the number of animals that can be sampled and the feasibility of repeated measurements. Blood samples serve as an effective surrogate tissue for transcriptomic analyses as reported in recent studies. Basu et al. (2021) have demonstrated that gene expression patterns in blood can detect transcriptomic profiles in other tissues, highlighting a functional link between circulating blood cells and systemic biological processes. The primary objective of the current study was to use blood analysis to investigate the genetic architecture underlying key FE traits including DMI_absolute_, DMI_adjusted_, ADG, and RFI. Multiple analytical approaches, including DGE analysis, WGCNA, and cis-eQTL mapping were integrated to identify blood-based gene signatures and regulatory networks with potential for application in multi-trait genetic selection strategies aimed at improving FE.

A subset of genes was identified to be overlapping from three analytical methods highlighting candidate FE regulators associated not just with differences in trait variations but also forming part of a coordinated biological network confirming functional importance. These genes include *DNMT3A, KANSL1, NCOR1, ACOX2, FANCF, CIMIP2B, LOC101115106, ARMH2, LOC132657496*, and *LOC114114576* for DMI_adjusted_, ADG, and RFI traits respectively representing high-confidence candidates for FE-based selection programmes. The absence of overlapping genes for DMI_absolute_ is likely due to its genetic architecture which may be influenced by behavioral and environmental factors diminishing clear genetic signals. Across the DMI_adjusted_ trait, three candidate overlapping genes (*DNMT3A, KANSL1,* and *NCOR1*) pointed towards a shared regulatory mechanism centred on partitioning of nutrients and chromatin dynamics. *DNMT3A,* which is an important epigenetic regulator encoding DNA methyltransferase, was found to be upregulated in this study (from DGE and WGCNA). Its involvement in cell proliferation, adipogenesis and muscle development may suggest its role in regulating tissue composition and growth, which may in turn influence energy utilisation and feed intake (Schinckel et al., 1998; Qimuge et al., 2019). The upregulated *KANSL1* encodes KAT8 regulatory NSL complex subunit 1 (Fejzo et al., 2021) that is involved in regulation of immune responses, organ development, and cell proliferation as reported in studies on chicken and dairy cows (Liu et al., 2024; Siberski-Cooper et al., 2024), suggesting a potential role of *KANSL1* in connecting DMI to immune-associated gene controls. *NCOR1* likely acts by influencing nutrient usage in a way that nutrients are used for lean growth rather than fat deposition, thereby improving overall FE of animal (Mirzapour et al., 2023). Collectively, these observations suggest that variations in DMI when adjusted for the animal’s body size arises from an interplay between chromatin dynamics, metabolic signalling, and immune-related pathways, highlighting the importance of epigenetic machinery in modulating feed-intake phenotypes. For ADG, 6 overlapping genes (*ACOX2, FANCF, CIMIP2B, ARMH2, LOC101115106, LOC132657496*) emerged, highlighting involvement in fatty acid oxidation, DNA repair, cell development and sperm functionality. *ACOX2* encodes a peroxisomal enzyme involved in the β-oxidation of branched chain fatty acids (Sharma and Hiller, 2018) with major roles in lipid metabolism and regulation of homeostasis (Wanders et al., 2001; Cao et al., 2025). In the present study, *ACOX2* expression was downregulated in association with increasing ADG suggesting a shift in energy metabolism associated with improved growth performance. Reduced *ACOX2* expression may reflect an altered lipid utilisation pattern that favours tissue accretion and growth over catabolism of fatty acids. Consistent with this, a recent study on beef cattle reported reduced liver *ACOX2* expression further inhibiting fatty acid oxidation thereby facilitating a metabolic environment supporting intramuscular fat deposition (Wang et al., 2025). Upregulation of *FANCF* observed in this study suggests an improved capacity of DNA repair (Papadaki et al., 2025) in fast growing lambs, which may also help in renewal of cells (including muscle cells) and tissue growth, matching the heavier building needs observed in fast-growing animals. The associations of *CIMIP2B* (upregulated) and *ARMH2* (downregulated), although not previously linked to growth in livestock, suggest additional cellular pathways contributing to cellular health, development and reproduction, likely reflecting a shift in energy partitioning towards fast growth. The two uncharacterised ADG-associated loci (*LOC101115106, LOC132657496*) may represent novel components of growth regulation, warranting further functional investigation. For the RFI trait, only one gene, *LOC114114576*, turned out to be overlapping from the three methods which regulates mitochondrial function by influencing permeability of mitochondria and homeostasis of reactive oxygen species (Zhou et al., 2024). Although no research demonstrates the function of this gene to livestock growth or RFI, the upregulation observed in this study suggests that changes in this gene activity may alter mitochondrial processes contributing to RFI, highlighting it as a candidate novel gene for future investigations.

While several genes were consistently identified across the three approaches, no genes overlapped across the four traits in the DGE analysis pointing to distinct gene activities linked to these trait variations. This suggests that even though these four traits are moderately correlated at the phenotypic level, they are separated by differences in their underlying biological processes at the molecular level emphasizing their biological diversity. Similar observations were reported in sheep as well as other livestock genomic and transcriptomic studies, with reported genetic correlations between growth and FE traits but these traits were regulated by different loci, genes or biological processes (Suárez-Vega et al., 2023; Schaub and Posbergh, 2025). For instance, a dairy sheep transcriptomic study linked FE to physiology of the mammary gland rather than growth associated pathways (Suárez-Vega et al., 2023). These findings show that phenotypic correlations might come from similar factors like intake and weight gain, but they differ in how the associated genes work, pointing to the varied and intricate nature of FE.

WGCNA provided a visual overview of how genes work together behind FE variations highlighting both shared and trait-dependent roles (Figure 2). For instance, the darkgrey gene module was negatively correlated with both DMI_absolute_ and ADG, meaning that genes in this module may be involved in processes that restricts both nutrient uptake and weight gain. This was supported by the functional enrichment analysis where Toll-like receptors, NF-κB activity and lipid metabolic pathways were enriched suggesting that immune and inflammatory responses play a major role in feed intake and weight gain pointing towards a connection where the animal’s defense system might shape how well it uses food and directs energy. Consistent with the earlier findings in this study showing the highest number of DEGs and overlapping genes across the three analytical approaches, ADG again emerged as the trait with the highest number of genes associated, dispersed in 5 WGCNA modules, showing the involvement of multiple biological layers influencing weight gain. Positive correlation of lightcyan and cyan modules with DMI_*adjusted*_ and RFI points to the dual association of genes influencing both traits. Genes in these modules were enriched in signal transduction, endocrine and immune system-related pathways indicating the involvement of endocrine and immune signalling pathways in regulating feed intake independent of body size and RFI. Similar findings were observed from the literature where endocrine and immune pathways were associated with RFI and FE (Ferronato et al., 2024). Interestingly, the lightgreen module exhibited a different association pattern. Genes in the lightgreen module were positively correlated with ADG but negatively correlated with RFI suggesting that their increased gene activity might result in increased weight gain whilst improving FE (as RFI would be decreased). Enriched pathways for the genes in this module (oxidative phosphorylation, RNA degradation and autophagy) shows an important biological link where growth aligns with better FE. Therefore, genes in this lightgreen module gives clues about how animals grow quicker while still using energy efficiently, suggesting possible routes for selective breeding.

While no genes passed the strict FDR threshold of 0.05 from the gene-level cis-eQTL analysis, likely due to the limited sample size, candidate eGenes (*n* = 280) identified in the current study are still potentially valid genes whose occurrence does not happen by chance. In particular, eGenes were selected based on permutation derived empirical *P* < 0.03, which reflects reproducibility under null distribution. The concordance of a subset of these eGenes between DGE and WGCNA suggest that they are not only regulated genetically but also show coordinated patterns of expression and trait associations making it highly unlikely that they were identified by chance.

Although blood is a readily accessible sample source and has been utilised in livestock studies investigating FE traits such as RFI and feed conversion ratio (FCR) (Jégou et al., 2016; Liu et al., 2016; Messad et al., 2021; Taiwo et al., 2022), the blood transcriptome primarily reflects circulating leukocytes and immune-associated processes. In contrast, biological mechanisms underlying FE are mostly driven by metabolic processes taking place in tissues such as liver, adipose tissue, skeletal muscle and gastrointestinal tract. Consequently, candidate genes identified in this study should be interpreted as blood-derived indicators of host physiological responses associated with FE, rather than direct regulators of growth and nutrient utilisation within these tissues. Nevertheless, published evidence suggests the potential of blood transcriptomic profiles to be useful in capturing biologically meaningful variations associated with FE traits, supporting their application as a minimally invasive approach for FE-associated candidate gene detection. Integrating blood transcriptomics with other tissue-specific gene expression data will be valuable for validating the functional relevance of candidate FE-associated genes identified here.

As the primary aim of this study was to identify candidate genes significantly associated with continuous variation in FE traits, DGE analysis was initially performed using each phenotype as a continuous explanatory variable. Although study lambs received different levels of microalgae oil, neither FE phenotypes nor global transcriptomic profiles showed clear separation among dietary groups. Nevertheless, sensitivity analyses using microalgae oil inclusion as a covariate showed that a proportion of the observed significant gene associations were influenced by microalgae oil, whereas several genes, particularly those identified for DMI_absolute_, DMI_adjusted_ and ADG, remained consistent across both models. These findings suggest that the reported candidate genes capture both robust phenotype-associated transcriptional signals and biological responses that may be shared between microalgae oil and FE. Therefore, multi-trait candidate genes identified through concordance between DGE, WGCNA, and cis-eQTL mapping methods should be interpreted within the context of the study design and warrant validation in larger independent populations.

This study has combined DGE, WGCNA, and cis-eQTL mapping methods to explore the genetic architecture behind FE traits in lambs. Key genes such as *DNMT3A, KANSL1, NCOR1, ACOX2, FANCF, CIMIP2B, LOC101115106, ARMH2, LOC132657496* and *LOC114114576* with repeated identification across the three methods points to their importance beyond statistical associations to biological controllers enclosed within coordinated networks. Although DMI_absolute_, DMI_adjusted_, ADG, and RFI were moderately correlated traits at the phenotypic level, a lack of overlapping DEGs suggest distinct molecular mechanisms underlying each trait such as neuroendocrine pathways, chromatin remodelling, developmental and immune response-associated pathways. Identification of trait-associated gene modules functionally enriched in signal transduction, oxidative phosphorylation, autophagy and RNA degradation further highlights that FE is driven by multiple biological mechanisms that are context-dependent. Findings suggest that animals can grow quicker without compromising FE while also highlighting how immune and endocrine processes redirect nutrients away from growth processes thereby reducing nutrient utilization. The discovery of several novel uncharacterized ADG- and RFI-associated loci such as *LOC101115106, LOC132657496,* and *LOC114114576* highlights the importance of further research in annotating and functionally validating the functions of these genes. While the identified candidate genes provide valuable insights into the potential regulatory mechanisms underlying FE traits, experimental validation was beyond the scope of the present study. These aspects will be addressed in future investigations to further substantiate and refine the gene-FE trait associations reported here. Collectively, results from this study show the importance of applying different analytical approaches to identifying critical genes and revealing species-specific regulatory divergence which can act as a foundation within breeding strategies. Incorporating the identified overlapping genes as candidate selection signatures in future breeding programs can help in selecting animals that grow faster and utilize nutrients more efficiently thereby reducing feeding costs, environmental emissions and enhance sustainability of modern farming practices.

## Data Availability

The original contributions presented in the study are publicly available. This data can be found in the NCBI Sequence Read Archive (SRA) repository with the accession numbers PRJNA1451055 and PRJNA1485014, at: https://www.ncbi.nlm.nih.gov/bioproject/?term=PRJNA1451055, https://www.ncbi.nlm.nih.gov/bioproject/?term=PRJNA1485014.
