# Supplementary material for: Genome-wide cis-expression Quantitative Trait Loci (eQTL) and transcriptomic signals reveal distinct molecular regulation across correlated feed efficiency traits
Source: Front Genet. 2026 Aug 21;17:1871055. doi: 10.3389/fgene.2026.1871055 (PMC13541292; doi:10.3389/fgene.2026.1871055)
Supplement: Supplementary file 1 [file DataSheet1.docx]

**Supplementary Files**

**Supplementary Tables**

Supplementary Table S1: Total reads, mapping quality and features assigned for lambs in the study generated using MultiQC software.

| **Sample Name** | **Percent of Assigned features** | **Percent of reads aligned** | **Total Reads in Millions** | **Connectivity (Z.k)** |
| --- | --- | --- | --- | --- |
| 6764 | 75.00% | 93.40% | 35 | -2.03 |
| 6768 | 76.10% | 93.50% | 33.1 | -0.10 |
| 6856 | 76.20% | 93.20% | 34 | -0.75 |
| 6865 | 73.40% | 92.80% | 32.6 | 0.64 |
| 6908 | 75.70% | 93.10% | 35.1 | 0.32 |
| 6914 | 76.70% | 93.10% | 32.9 | 0.19 |
| 6960 | 77.20% | 93.50% | 32.5 | -0.82 |
| 6968 | 77.50% | 92.90% | 32.5 | -0.90 |
| 6973 | 71.40% | 94.80% | 31.1 | 0.38 |
| 6976 | 74.30% | 93.20% | 28 | -0.74 |
| 6977 | 69.90% | 91.80% | 27.2 | -2.91 |
| 7025 | 73.40% | 93.80% | 29 | 0.28 |
| 7033 | 63.40% | 81.80% | 28.7 | 0.31 |
| 7050 | 69.40% | 87.60% | 31.2 | 0.51 |
| 7059 | 73.30% | 94.20% | 29 | 1.27 |
| 7064 | 73.20% | 94.50% | 26.5 | 0.66 |
| 7066 | 74.30% | 94.40% | 25.6 | 0.45 |
| 7068 | 72.70% | 93.60% | 25.5 | 1.16 |
| 7070 | 63.50% | 81.70% | 25 | 0.43 |
| 7072 | 50.10% | 63.00% | 25.9 | - |
| 7073 | 1.30% | 1.80% | 24.3 | - |
| 7085 | 1.50% | 2.00% | 27.1 | - |
| 7093 | 68.90% | 87.40% | 28 | 1.14 |
| 7094 | 71.70% | 93.70% | 28.5 | -2.37 |
| 7097 | 74.20% | 94.30% | 27.2 | 0.33 |
| 7105 | 71.60% | 93.50% | 24.4 | 0.53 |
| 7115 | 71.30% | 93.20% | 27.6 | 0.33 |
| 7117 | 72.70% | 93.70% | 25.8 | 0.12 |
| 7133 | 71.60% | 92.10% | 30.3 | 0.58 |
| 7144 | 63.50% | 83.80% | 25.2 | -0.19 |
| 7153 | 72.50% | 91.70% | 24.8 | -0.56 |
| 7184 | 73.10% | 94.00% | 24.2 | -0.16 |
| 7196 | 76.10% | 93.40% | 29.9 | -0.99 |
| 7198 | 72.50% | 92.90% | 27.4 | 1.05 |
| 7220 | 72.80% | 93.20% | 30.6 | 1.01 |
| 7236 | 72.40% | 93.10% | 31.2 | 0.79 |

Supplementary Table S2: Significant differentially expressed genes (DEGs) associated with feed efficiency (FE) traits; absolute dry matter intake (DMI_absolute_), DMI_adjusted_, average daily live weight gain (ADG), and residual feed intake (RFI) in studied lambs. These genes were extracted based on the *adjusted P* cut-off less than 0.05 and |Log_2_ fold change| of 2.

| **Dry Matter Intake-Absolute (DMIabsolute)** | | | |
| --- | --- | --- | --- |
| **Gene ID** | **Log_2_ fold change** | **Raw *P*** | **adjusted *P*** |
| *RGS2* | -2.56 | 9.11E-11 | 1.35E-06 |
| *CD99* | 5.23 | 4.09E-05 | 0.04 |
| **Dry Matter Intake-Adjusted for body size (DMI_adjusted_)** | | | |
| **Gene ID** \| **Original Log_2_ fold change (phenotype-range adjusted)** \| **Raw *P*** \| **adjusted *P*** | | | |
| *PUM1* | 31.81 (0.286) | 6.53E-06 | 0.0090 |
| *ARID1A* | 40.10 (0.360) | 2.62E-05 | 0.0114 |
| *BRD4* | 41.26 (0.373) | 2.78E-05 | 0.0114 |
| *MTOR* | 39.11 (0.351) | 3.44E-05 | 0.0114 |
| *BAZ1B* | 39.38 (0.354) | 4.15E-05 | 0.0114 |
| *WNK1* | 38.70 (0.348) | 8.40E-05 | 0.0129 |
| *NCOR1* | 25.63 (0.230) | 8.17E-05 | 0.0129 |
| *DYNC1H1* | 43.07 (0.387) | 5.76E-05 | 0.0129 |
| *SRRM2* | 37.90 (0.341) | 8.43E-05 | 0.0129 |
| *GLG1* | 41.79 (0.376) | 9.86E-05 | 0.0135 |
| *LOC101111060* | 37.80 (0.340) | 0.000262222 | 0.0227 |
| *CNTRL* | 35.58 (0.320) | 0.000292189 | 0.0227 |
| *DNMT3A* | 39.83 (0.358) | 0.000203539 | 0.0227 |
| *ZZEF1* | 32.73 (0.294) | 0.000297562 | 0.0227 |
| *KANSL1* | 26.15 (0.235) | 0.000265976 | 0.0227 |
| *ASXL1* | 35.65 (0.320) | 0.000276504 | 0.0227 |
| *CREBBP* | 37.23 (0.335) | 0.000205342 | 0.0227 |
| *TRRAP* | 36.89 (0.332) | 0.000260427 | 0.0227 |
| *PRRC2B* | 29.91 (0.269) | 0.000445572 | 0.0322 |
| *EP400* | 26.50 (0.238) | 0.000547153 | 0.0375 |
| *ACIN1* | 25.54 (0.229) | 0.000580671 | 0.0379 |
| *TCF20* | 34.53 (0.310) | 0.000634338 | 0.0396 |
| *AKAP13* | 34.02 (0.306) | 0.000800672 | 0.0458 |
| *SRCAP* | 33.75 (0.303) | 0.000800956 | 0.0458 |
| **Average daily live weight gain (ADG)** | | | |
| **Gene ID** | **Log2 fold change** | **Raw *P*** | **Adjusted *P*** |
| *LOC132657496* | -18.90 | 1.68E-05 | 0.0152 |
| *LOC121818840* | -11.94 | 5.67E-05 | 0.0282 |
| *LOC114118728* | -11.30 | 9.18E-05 | 0.0322 |
| *LOC105601985* | -9.22 | 7.39E-05 | 0.0321 |
| *LOC132659398* | -9.22 | 0.000271998 | 0.0482 |
| *LOC132657619* | -8.79 | 3.86E-06 | 0.0068 |
| *GRPR* | -8.70 | 0.000178217 | 0.0383 |
| *LOC132659423* | -8.22 | 2.94E-06 | 0.0068 |
| *CDC42BPB* | -8.18 | 3.51E-06 | 0.0068 |
| *LOC121820454* | -7.42 | 0.000294784 | 0.0497 |
| *LOC132658274* | -7.42 | 0.000117446 | 0.0347 |
| *LOC121817654* | -7.09 | 0.000226277 | 0.0434 |
| *CLEC1A* | -7.00 | 1.06E-05 | 0.0129 |
| *LOC132657507* | -6.97 | 0.000109411 | 0.0347 |
| *LOC121816932* | -6.90 | 0.000239298 | 0.0449 |
| *LOC114110470* | -6.46 | 0.000123676 | 0.0353 |
| *FNDC3B* | -6.43 | 2.82E-07 | 0.0022 |
| *APH1B* | -6.42 | 3.86E-06 | 0.0068 |
| *LOC121820423* | -6.39 | 0.000143945 | 0.0353 |
| *LOC121816934* | -5.91 | 9.51E-05 | 0.0322 |
| *LOC121819237* | -5.86 | 0.000151313 | 0.0353 |
| *MCTP2* | -5.63 | 2.45E-05 | 0.0186 |
| *KLHL8* | -5.56 | 2.19E-05 | 0.0174 |
| *LOC101107489* | -5.51 | 9.08E-05 | 0.0322 |
| *MXD1* | -5.43 | 5.57E-05 | 0.0282 |
| *LOC114115018* | -5.41 | 0.000213604 | 0.0425 |
| *LOC105611231* | -5.33 | 4.09E-05 | 0.0257 |
| *LOC132657337* | -5.30 | 0.000257339 | 0.0466 |
| *LOC132658623* | -5.29 | 4.10E-05 | 0.0257 |
| *LOC132658352* | -5.08 | 8.75E-05 | 0.0322 |
| *RBM47* | -5.08 | 1.18E-05 | 0.0135 |
| *RNF24* | -4.82 | 9.13E-05 | 0.0322 |
| *LOC101115106* | -4.80 | 5.28E-05 | 0.0280 |
| *LOC121819274* | -4.72 | 5.51E-06 | 0.0080 |
| *SLC26A8* | -4.70 | 7.28E-05 | 0.0321 |
| *WDFY3* | -4.69 | 0.000156092 | 0.0355 |
| *LOC114115024* | -4.51 | 7.99E-05 | 0.0322 |
| *LOC106990930* | -4.22 | 0.00021898 | 0.0430 |
| *MB21D2* | -4.20 | 0.000167693 | 0.0366 |
| *GK* | -4.14 | 0.000294112 | 0.0497 |
| *ATXN1* | -4.09 | 2.95E-06 | 0.0068 |
| *IGSF6* | -4.07 | 0.000290737 | 0.0497 |
| *LOC105606185* | -4.03 | 0.000221448 | 0.0430 |
| *LOC132659414* | -4.00 | 0.00018864 | 0.0390 |
| *LOC132660131* | -3.91 | 4.20E-05 | 0.0257 |
| *SLC6A20* | -3.75 | 1.72E-07 | 0.0022 |
| *ACOX2* | -3.75 | 0.000167787 | 0.0366 |
| *ST7L* | -3.69 | 0.000141498 | 0.0353 |
| *DIXDC1* | -3.68 | 0.000111878 | 0.0347 |
| *UBXN2B* | -3.62 | 9.33E-05 | 0.0322 |
| *ISM2* | -3.48 | 2.15E-05 | 0.0174 |
| *ECT2* | -3.46 | 0.000137234 | 0.0353 |
| *RNF149* | -3.27 | 0.000251763 | 0.0466 |
| *TMEM216* | -3.07 | 0.000150256 | 0.0353 |
| *LOC114116893* | -3.05 | 0.000265356 | 0.0475 |
| *FBXL5* | -2.94 | 1.33E-05 | 0.0141 |
| *ARMH2* | -2.89 | 0.000132874 | 0.0353 |
| *PICALM* | -2.74 | 0.000147881 | 0.0353 |
| *FGGY* | -2.73 | 0.000138398 | 0.0353 |
| *ANKFY1* | -2.64 | 4.67E-05 | 0.0266 |
| *LOC105608012* | -2.42 | 0.000115921 | 0.0347 |
| *LOC132657287* | -2.32 | 0.000136809 | 0.0353 |
| *INSYN2B* | -2.31 | 0.000232884 | 0.0442 |
| *ANO10* | -2.15 | 4.62E-05 | 0.0266 |
| *KHDC4* | -2.12 | 2.83E-05 | 0.0205 |
| *PSENEN* | 2.02 | 8.36E-05 | 0.0322 |
| *MFSD5* | 2.24 | 0.000153 | 0.0353 |
| *BLOC1S3* | 2.41 | 1.62E-06 | 0.0068 |
| *UROS* | 3.09 | 8.64E-06 | 0.0115 |
| *COMMD2* | 3.11 | 0.000180224 | 0.0383 |
| *MAGEF1* | 3.89 | 1.72E-05 | 0.0152 |
| *TMEM250* | 3.92 | 3.40E-05 | 0.0235 |
| *ID3* | 3.95 | 0.000208236 | 0.0420 |
| *FANCF* | 4.49 | 0.000114594 | 0.0347 |
| *LOC114108769* | 4.89 | 0.000280979 | 0.0492 |
| *NHLRC1* | 5.62 | 6.95E-05 | 0.0321 |
| *IGFBP4* | 6.21 | 0.000186265 | 0.0390 |
| *FAT2* | 6.90 | 0.000131392 | 0.0353 |
| *LOC132660167* | 8.62 | 0.000100205 | 0.0326 |
| *HRH4* | 10.20 | 0.000127424 | 0.0353 |
| *SCN3B* | 10.67 | 0.000166885 | 0.0366 |
| *CIMIP2B* | 11.25 | 1.70E-05 | 0.0152 |
| *SNAP25* | 12.70 | 4.65E-06 | 0.0074 |
| *LOC101104522* | 13.45 | 0.000144655 | 0.0353 |
| *PPP2R2B* | 18.92 | 8.18E-05 | 0.0322 |
| **Residual feed intake (RFI)** | | | |
| **Gene ID** | **Log2 fold change** | **Raw *P*** | **Adjusted *P*** |
| *CD24* | -2.99 | 3.89E-07 | 0.0007 |
| *ACTRT3* | -3.51 | 2.52E-06 | 0.0028 |
| *LOC114114576* | 2.56 | 4.26E-06 | 0.0035 |
| *LOC101109907* | 2.04 | 0.000371186 | 0.0349 |

Supplementary Table S3: Overlap of differentially expressed genes (DEGs) identified from DESeq2 models with and without inclusion of microalgae oil as a covariate.

| **DMI_absolute_** | | | |
| --- | --- | --- | --- |
| **Overlapped Gene ID** | **Log2 fold change** | **Raw *P*** | **Adjusted *P*** |
| *RGS2* | -2.181 | 1.47E-07 | 0.001 |
| **DMI_adjusted_** | | | |
| **Overlapped Gene ID** | **Original Log_2_ fold change (phenotype-range adjusted)** | **Raw *P*** | **Adjusted *P*** |
| *PUM1* | 31.36 (0.28) | 7.63E-05 | 0.04 |
| *BRD4* | 37.15 (0.33) | 0.0001 | 0.04 |
| *MTOR* | 37.39 (0.34) | 0.0001 | 0.04 |
| *CNTRL* | 40.09 (0.36) | 9.00E-05 | 0.04 |
| *ACIN1* | 29.32 (0.26) | 1.27E-05 | 0.02 |
| **ADG** | | | |
| **Overlapped Gene ID** | **Log2 fold change** | **Raw *P*** | **Adjusted *P*** |
| *ANKFY1* | -2.51 | 5.80E-05 | 0.034 |
| *ANO10* | -2.16 | 7.87E-05 | 0.037 |
| *APH1B* | -6.12 | 1.46E-05 | 0.018 |
| *ATXN1* | -4.01 | 1.22E-06 | 0.004 |
| *BLOC1S3* | 2.13 | 1.17E-05 | 0.018 |
| *CDC42BPB* | -9.30 | 2.44E-07 | 0.002 |
| *CIMIP2B* | 12.01 | 1.62E-05 | 0.019 |
| *CLEC1A* | -6.55 | 7.47E-05 | 0.037 |
| *COMMD2* | 3.42 | 8.10E-05 | 0.037 |
| *DIXDC1* | -3.88 | 4.59E-05 | 0.030 |
| *FBXL5* | -2.48 | 0.000112 | 0.040 |
| *FNDC3B* | -6.39 | 8.85E-08 | 0.002 |
| *KLHL8* | -5.92 | 4.10E-06 | 0.010 |
| *LOC101107489* | -5.69 | 4.20E-05 | 0.030 |
| *LOC105601985* | -9.36 | 0.000106 | 0.040 |
| *LOC106990930* | -4.30 | 4.54E-05 | 0.030 |
| *LOC114108769* | 5.38 | 9.27E-05 | 0.037 |
| *LOC114115018* | -5.69 | 7.62E-05 | 0.037 |
| *LOC114118728* | -11.75 | 6.26E-05 | 0.034 |
| *LOC121816932* | -6.70 | 8.20E-05 | 0.037 |
| *LOC121816934* | -5.68 | 9.37E-05 | 0.037 |
| *LOC121818840* | -13.41 | 1.32E-05 | 0.018 |
| *LOC121819237* | -6.09 | 4.76E-05 | 0.030 |
| *LOC121819274* | -4.75 | 9.07E-06 | 0.018 |
| *LOC132657496* | -18.44 | 0.000108 | 0.040 |
| *LOC132657619* | -8.70 | 8.67E-07 | 0.004 |
| *LOC132658352* | -5.12 | 3.41E-05 | 0.028 |
| *LOC132659423* | -8.57 | 5.86E-06 | 0.013 |
| *MAGEF1* | 3.34 | 0.000127 | 0.044 |
| *MCTP2* | -5.48 | 3.77E-05 | 0.029 |
| *MXD1* | -5.06 | 4.87E-05 | 0.030 |
| *RBM47* | -5.01 | 1.13E-05 | 0.018 |
| *RNF24* | -4.94 | 3.36E-05 | 0.028 |
| *SCN3B* | 11.82 | 2.70E-05 | 0.028 |
| *SLC6A20* | -3.60 | 3.79E-07 | 0.002 |
| *SNAP25* | 13.42 | 2.24E-06 | 0.007 |
| *UROS* | 2.76 | 9.53E-05 | 0.037 |

Supplementary Table S4: Estimates of soft threshold powers obtained from WGCNA. The scale-free topology indices (SFT.R.sq), slope, and mean connectivity (Mean.k) across different power values ranging from 1 to 15 are tabulated. The power value with maximum SFT.R.sq and minimum mean connectivity was chosen (power =4) as the power threshold.

| **Power** | **SFT.R. Sq** | **Slope** | **Truncated R. sq** | **Mean.k** | **Median.k** | **Max.k** |
| --- | --- | --- | --- | --- | --- | --- |
| 1 | 0.46 | -2.69 | 0.91 | 3681.18 | 3.542812e+03 | 5912.52 |
| 2 | 0.78 | -2.44 | 0.97 | 1077.43 | 9.599581e+02 | 2653.40 |
| 3 | 0.87 | -2.32 | 0.98 | 400.98 | 3.204876e+02 | 1452.94 |
| 4 | 0.89 | -2.22 | 0.98 | 175.08 | 1.218796e+02 | 896.25 |
| 5 | 0.88 | -2.17 | 0.98 | 85.89 | 5.112163e+01 | 597.70 |
| 6 | 0.88 | -2.11 | 0.98 | 46.07 | 2.302454e+01 | 419.97 |
| 7 | 0.87 | -2.09 | 0.98 | 26.51 | 1.102884e+01 | 306.69 |
| 8 | 0.87 | -2.06 | 0.98 | 16.15 | 5.542549e+00 | 230.73 |
| 9 | 0.88 | -2.01 | 0.99 | 10.30 | 2.884856e+00 | 177.75 |
| 10 | 0.89 | -1.96 | 0.99 | 6.83 | 1.563524e+00 | 139.61 |
| 11 | 0.88 | -1.94 | 0.99 | 3.30 | 5.017343e-01 | 90.20 |
| 12 | 0.89 | -1.88 | 1.00 | 1.75 | 1.782936e-01 | 61.13 |
| 13 | 0.90 | -1.86 | 1.00 | 1.01 | 6.826248e-02 | 42.97 |
| 14 | 0.89 | -1.83 | 0.98 | 0.61 | 2.782033e-02 | 31.10 |
| 15 | 0.90 | -1.79 | 0.97 | 0.39 | 1.194695e-02 | 23.04 |

Supplementary Table S5: Overlapping genes from WGCNA and DGE analysis for the studied FE traits.

| **Phenotype_WGCNA vs Phenotype_DGE** | **Number of overlapping genes** | **Overlapping genes** |
| --- | --- | --- |
| DMI_absolute__WGCNA  Vs DMI__absolute__DGE | *0* |  |
| DMI__adjusted__WGCNA vs DMI__adjusted__DGE | *10* | *NCOR1, PUM1, GLG1, BAZ1B, AKAP13, LOC101111060, DNMT3A, KANSL1, MTOR, EP400* |
| ADG_WGCNA vs ADG_DGE | *66* | *LOC132659423, ATXN1, LOC121819274, APH1B, ANKFY1, LOC132658274, KLHL8, RBM47, MCTP2, LOC132657619, CLEC1A, MXD1, DIXDC1, LOC121816934, MB21D2, RNF149, LOC121818840, IGSF6, LOC121816932, RNF24, GK, LOC106990930, LOC101107489, WDFY3, LOC114110470, LOC105601985, LOC132657337, LOC121820454, LOC121819237, LOC121817654, GRPR, LOC132657496, TMEM216, CDC42BPB, ANO10, LOC132657287, LOC101115106, LOC132659414, LOC105608012, ARMH2, LOC105606185, ACOX2, INSYN2B, LOC121820423, LOC114118728, LOC132659398, SLC6A20, FNDC3B, KHDC4, FGGY, LOC132658623, PICALM, UBXN2B, LOC105611231, ST7L, LOC114116893, LOC132657507, LOC132658352, LOC114115018, CIMIP2B, FANCF, PSENEN, NHLRC1, COMMD2, IGFBP4, ID3* |
| RFI_WGCNA vs RFI_DGE | *3* | *LOC114114576, ACTRT3, CD24* |

Supplementary Table S6: Top enriched KEGG pathways in the WGCNA modules associated with the four studied traits.

| **Module** | **KEGG ID** | **Description** | **Adjusted *P*** | **Gene counts** |
| --- | --- | --- | --- | --- |
| Black | oas04668 | TNF signaling pathway | 4.29E-05 | 27 |
| Black | oas04151 | PI3K-Akt signaling pathway | 4.73E-05 | 57 |
| Black | oas04630 | JAK-STAT signaling pathway | 5.75E-05 | 37 |
| Black | oas04620 | Toll-like receptor signaling pathway | 0.00018222 | 24 |
| Black | oas05321 | Inflammatory bowel disease | 0.00035177 | 16 |
| Black | oas04010 | MAPK signaling pathway | 0.00037402 | 44 |
| Black | oas04820 | Cytoskeleton in muscle cells | 0.00053779 | 36 |
| Black | oas04625 | C-type lectin receptor signaling pathway | 0.00075701 | 21 |
| Black | oas04659 | Th17 cell differentiation | 0.00075701 | 21 |
| Black | oas04066 | HIF-1 signaling pathway | 0.00103915 | 22 |
| Black | oas04910 | Insulin signaling pathway | 0.00217758 | 24 |
| Black | oas04150 | mTOR signaling pathway | 0.00633618 | 25 |
| Black | oas04068 | FoxO signaling pathway | 0.00676196 | 21 |
| Black | oas04920 | Adipocytokine signaling pathway | 0.00694604 | 14 |
| Black | oas04072 | Phospholipase D signaling pathway | 0.00694604 | 23 |
| Black | oas04658 | Th1 and Th2 cell differentiation | 0.00870626 | 16 |
| Black | oas04935 | Growth hormone synthesis, secretion and action | 0.01108917 | 19 |
| Black | oas04330 | Notch signaling pathway | 0.01270216 | 12 |
| Black | oas05145 | Toxoplasmosis | 0.01349557 | 20 |
| Black | oas04662 | B cell receptor signaling pathway | 0.02664177 | 13 |
| Black | oas01522 | Endocrine resistance | 0.02664177 | 15 |
| Black | oas03320 | PPAR signaling pathway | 0.03469847 | 13 |
| Darkgrey | oas04620 | Toll-like receptor signalling pathway | 0.00103645 | 6 |
| Darkgrey | oas04064 | NF-kappa B signaling pathway | 0.00318642 | 5 |
| Darkgrey | oas04621 | NOD-like receptor signaling pathway | 0.01890777 | 5 |
| Lightgreen | oas00190 | Oxidative phosphorylation | 1.55E-09 | 49 |
| Lightgreen | oas05415 | Diabetic cardiomyopathy | 3.65E-08 | 54 |
| Lightgreen | oas04932 | Non-alcoholic fatty liver disease | 3.64E-05 | 41 |
| Lightgreen | oas01232 | Nucleotide metabolism | 0.00049358 | 21 |
| Lightgreen | oas03008 | Ribosome biogenesis in eukaryotes | 0.00162772 | 26 |
| Lightgreen | oas03082 | ATP-dependent chromatin remodelling | 0.00418142 | 24 |
| Lightgreen | oas03018 | RNA degradation | 0.02382438 | 17 |
| Lightgreen | oas00240 | Pyrimidine metabolism | 0.02626061 | 13 |
| Blue | oas03018 | RNA degradation | 0.01769757 | 20 |
| Blue | oas04110 | Cell cycle | 0.0185985 | 32 |
| Blue | oas04120 | Ubiquitin mediated proteolysis | 0.04591746 | 29 |
| Lightcyan | oas04015 | Rap1 signaling pathway | 0.00046752 | 15 |
| Lightcyan | oas04621 | NOD-like receptor signaling pathway | 0.01729173 | 11 |
| Cyan | oas04660 | T cell receptor signaling pathway | 0.02467458 | 9 |
| Cyan | oas04931 | Insulin resistance | 0.02467458 | 8 |
| Purple | oas04660 | T cell receptor signaling pathway | 0.00090478 | 16 |
| Purple | oas04659 | Th17 cell differentiation | 0.00139375 | 14 |
| Purple | oas04072 | Phospholipase D signaling pathway | 0.0061028 | 15 |
| Purple | oas04658 | Th1 and Th2 cell differentiation | 0.00826978 | 11 |
| Purple | oas04514 | Cell adhesion molecule (CAM) interaction | 0.01958622 | 14 |
| Purple | oas04611 | Platelet activation | 0.01958622 | 12 |
| Purple | oas04014 | Ras signaling pathway | 0.01958622 | 18 |
| Purple | oas05340 | Primary immunodeficiency | 0.01958622 | 6 |
| Purple | oas00565 | Ether lipid metabolism | 0.02548686 | 7 |
| Purple | oas04015 | Rap1 signaling pathway | 0.03008903 | 16 |
| Purple | oas04923 | Regulation of lipolysis in adipocytes | 0.04011645 | 7 |
| Magenta | oas04662 | B cell receptor signaling pathway | 8.38E-07 | 17 |
| Magenta | oas04672 | Intestinal immune network for IgA production | 0.00011921 | 11 |
| Magenta | oas04640 | Hematopoietic cell lineage | 0.00041271 | 15 |
| Magenta | oas05340 | Primary immunodeficiency | 0.0019254 | 8 |
| Magenta | oas04064 | NF-kappa B signaling pathway | 0.00654371 | 13 |
| Magenta | oas04612 | Antigen processing and presentation | 0.01606797 | 11 |
| Magenta | oas04658 | Th1 and Th2 cell differentiation | 0.01606797 | 11 |
| Magenta | oas04514 | Cell adhesion molecule (CAM) interaction | 0.02361665 | 15 |
| Magenta | oas01232 | Nucleotide metabolism | 0.02378795 | 10 |
| Magenta | oas05321 | Inflammatory bowel disease | 0.04013837 | 8 |

Supplementary Table S7: Lead cis-eQTL single nucleotide polymorphisms (eSNPs, *n*=93*)*) identified after linkage disequilibrium (LD)-based clumping (r² = 0.8, 1 Mb window), showing associations with corresponding eGenes (*n*=74*)*. Reported values include false discovery rate (FDR) and effect size. Only SNPs meeting the FDR < 0.05 threshold are reported.

| **Lead Single nucleotide polymorphism (eSNPs)** | **eGenes** | **False discovery rate (FDR)** | **Effect size (beta)** |
| --- | --- | --- | --- |
| s04534.1 | *LOC132658291* | 1.443 x 10^-5^ | 0.8570 |
| s32787.1 | *TOE1* | 2.660 x 10^-5^ | 0.1947 |
| s71260.1 | *PLPPR1* | 0.0010 | 0.6693 |
| OAR11_23034869.1 | *CRK* | 0.0040 | -0.2957 |
| OAR25_8118133.1 | *B3GALNT2* | 0.0045 | 0.3891 |
| OAR14_51383053.1 | *LOC121816523* | 0.0051 | -0.6667 |
| OAR21_6678031.1 | *NOX4* | 0.0051 | 0.3388 |
| s00419.1 | *LOC114118725* | 0.0051 | -0.6486 |
| OAR10_17703888.1 | *RCBTB2* | 0.0057 | -0.3010 |
| OAR15_15729207.1 | *LOC114118382* | 0.0057 | 0.4676 |
| s22440.1 | *B3GALNT2* | 0.0057 | 0.4208 |
| s27840.1 | *LOC132660162* | 0.0057 | 0.3450 |
| DU362488_275.1 | *LOC105603087* | 0.0069 | 1.6576 |
| s25251.1 | *LOC105604626* | 0.0069 | -0.6577 |
| s74478.1 | *CHSY1* | 0.0075 | 0.6051 |
| OAR4_47543789.1 | *LOC132659683* | 0.0115 | 0.5743 |
| s51197.1 | *CRK* | 0.0116 | -0.3287 |
| s38265.1 | *LOC114113871* | 0.0116 | -0.6595 |
| OAR15_29161278.1 | *DSCAML1* | 0.0116 | 0.4997 |
| s06503.1 | *WIPF3* | 0.0116 | 0.3991 |
| s38693.1 | *LOC132658435* | 0.0116 | 0.5604 |
| OAR17_36154362.1 | *LOC105602806* | 0.0116 | 0.2579 |
| s33369.1 | *GLI4* | 0.0116 | -0.2842 |
| OAR4_66070878.1 | *LOC101109351* | 0.0116 | -0.4106 |
| OAR7_24958064.1 | *LOC101105064* | 0.0128 | 0.2915 |
| s07306.1 | *LOC121817511* | 0.0150 | -0.7494 |
| OAR21_1390902.1 | *LOC121817511* | 0.0150 | 0.7850 |
| OAR9_14653377.1 | *RHPN1* | 0.0150 | 0.2792 |
| s43564.1 | *BCKDHB* | 0.0151 | -0.1913 |
| OAR4_48471516.1 | *LOC132659683* | 0.0154 | 0.5042 |
| OAR8_74747120.1 | *LRP11* | 0.0207 | 0.3837 |
| OAR20_13743375.1 | *SAYSD1* | 0.0212 | 0.3063 |
| OAR14_65487938.1 | *LOC114118094* | 0.0215 | 0.4624 |
| OAR25_8059494.1 | *B3GALNT2* | 0.0226 | 0.3009 |
| OAR19_8504.1 | *URGCP* | 0.0226 | 0.1171 |
| OAR1_19817567.1 | *TOE1* | 0.0228 | 0.1923 |
| OAR1_166341363.1 | *SENP7* | 0.0228 | -0.2030 |
| OAR2_1189108.1 | *LOC121818569* | 0.0228 | -0.6115 |
| OAR14_1720811.1 | *VSTM2B* | 0.0228 | 0.6573 |
| OAR11_14457337.1 | *NLE1* | 0.0228 | -0.3067 |
| s10820.1 | *LOC101116324* | 0.0228 | 0.6395 |
| OAR18_22964031.1 | *HOMER2* | 0.0228 | 0.4949 |
| s30082.1 | *COL5A1* | 0.0228 | 0.8418 |
| OAR20_25488358.1 | *LOC105612706* | 0.0252 | 0.6832 |
| OAR9_30971182.1 | *HAS2* | 0.0316 | 0.4945 |
| s15165.1 | *LOC132658235* | 0.0316 | -0.2689 |
| s26017.1 | *LRP11* | 0.0317 | -0.5626 |
| OAR14_2924168.1 | *LOC121816457* | 0.0317 | 0.4423 |
| OAR6_58614245.1 | *RELL1* | 0.0319 | -0.2542 |
| OAR1_20007545.1 | *TOE1* | 0.0319 | 0.1731 |
| OAR20_9311938.1 | *TCP11* | 0.0319 | 0.2765 |
| OAR3_39102445.1 | *LOC121819269* | 0.0319 | -0.4106 |
| s39037.1 | *CFAP77* | 0.0319 | -0.2585 |
| OAR10_28087786.1 | *STARD13* | 0.0319 | 0.4065 |
| OAR17_5295579.1 | *TMEM131L* | 0.0319 | -0.1287 |
| s59500.1 | *LOC101117791* | 0.0320 | -0.3957 |
| OAR9_30983236.1 | *HAS2* | 0.0321 | 0.5486 |
| OAR1_20180147_X.1 | *BEST4* | 0.0324 | 0.4069 |
| OAR22_24607042.1 | *STN1* | 0.0329 | 0.2506 |
| OAR25_25639445.1 | *COL13A1* | 0.0335 | 0.4281 |
| s07104.1 | *GPR135* | 0.0336 | -0.3052 |
| s21252.1 | *URB2* | 0.0340 | -0.1272 |
| OAR14_49924498.1 | *SERTAD3* | 0.0340 | -0.1897 |
| OAR10_30038002.1 | *B3GLCT* | 0.0340 | -0.2852 |
| OAR1_20035449.1 | *BEST4* | 0.0369 | 0.3326 |
| OAR23_36925377.1 | *ENOSF1* | 0.0377 | -0.2172 |
| s63380.1 | *AGPAT4* | 0.0382 | 0.5265 |
| OAR1_19425357.1 | *TOE1* | 0.0391 | 0.1795 |
| s19581.1 | *URB2* | 0.0391 | 0.1303 |
| OAR14_48832510.1 | *LTBP4* | 0.0391 | 0.5154 |
| OAR9_32054016.1 | *HAS2* | 0.0391 | -0.5444 |
| OAR20_24357620.1 | *GSTA1-1* | 0.0391 | 0.6567 |
| OAR23_28704954.1 | *CDH2* | 0.0391 | -0.2795 |
| OAR10_27878618.1 | *STARD13* | 0.0391 | -0.3106 |
| OAR2_21602679.1 | *PLPPR1* | 0.0393 | -0.5645 |
| s72605.1 | *STARD13* | 0.0417 | 0.3184 |
| OAR7_58293730.1 | *GABPB1* | 0.0433 | -0.2177 |
| s24656.1 | *EXOSC6* | 0.0434 | -0.3770 |
| OAR14_52345906.1 | *LOC121816523* | 0.0434 | -0.6077 |
| OAR7_25090973.1 | *APEX1* | 0.0437 | -0.1960 |
| OAR1_31693459.1 | *PRKAA2* | 0.0439 | -0.4333 |
| s30544.1 | *RNF212* | 0.0439 | 0.3164 |
| s52270.1 | *MYH14* | 0.0443 | 0.7005 |
| s01893.1 | *LOC114117316* | 0.0471 | 0.2417 |
| s49885.1 | *CFAP77* | 0.0471 | 0.2433 |
| s22929.1 | *CRYL1* | 0.0471 | -0.9792 |
| OAR8_74299776.1 | *LOC114116193* | 0.0471 | 0.4747 |
| s15823.1 | *CCDC97* | 0.0471 | -0.2023 |
| s41927.1 | *ZNF34* | 0.0499 | -0.2440 |
| s01601.1 | *ACKR2* | 0.0499 | -0.2916 |
| OAR2_53359884.1 | *DNAJB5* | 0.0499 | -0.2653 |
| OAR1_19945652.1 | *TOE1* | 0.0499 | 0.1689 |
| s76002.1 | *MRPS2* | 0.0499 | -0.5185 |

Supplementary Table S8: Shared genes across WGCNA and cis-eQTL mapping for the four FE traits.

| **Trait** | **Module** | **Common genes from WGCNA and cis-eQTL** |
| --- | --- | --- |
| DMI_absolute_ | Darkgrey | No genes |
| DMIa_djusted_ | Lightcyan | *ABCA3, ACER2* |
| DMIa_djusted_ | Cyan | *COL5A1, RNF212, LOC114117316, SECISBP2L, LOC121819003, LOC121818811, LOC132660015* |
| DMIa_djusted_ | Purple | *LOC101116324, COL13A1, LOC114116895, SLC39A14, LOC132657946, ATP8B2, KBTBD11, LOC121817001, LOC114115895, WDR17, LOC121819423* |
| ADG | Black | *PLPPR1, CHSY1, LOC101109351, MYH14, CRYL1, DNAJB5, HIP1, DDX31, LOC105604793, PHLDB3, CELF6, LOC121818762, CLTA, LOC121817214, LOC132657299, CERS3, LOC105603000, PAQR5, LOC132657778, LOC101109370, KCP, OPA3, LOC132658234, UBE2QL1, LOC121816699, LOC101109890, LOC121817285, LOC114108871, LOC105616380, LOC132657496, LOC121816557* |
| ADG | Blue | *TOE1, LOC114113871, SAYSD1, SENP7, LOC121818569, HAS2, LOC132658235, LOC121816457, RELL1, TMEM131L, STN1, B3GLCT, AGPAT4, CDH2, IMPG2, ZNF34, ACKR2, ECPAS, ZFP1, LOC121817910, LOC121820187, PEX1, LOC121820473, PBRM1, TGFBR1, H3-4, UNC45B, TRNAC-GCA_60, CEP192, LOC114115267, ZC2HC1B, CCNT1, LOC106991816, GALK2, WFIKKN2, MAGI1, MMAA, LOC101120001, LOC101105609, PIGG, LOC121816747, LOC114110249, ZNF391* |
| ADG | Darkgrey | *SLC6A20, FNDC3B, KHDC4, FGGY, LOC132658623, PICALM, UBXN2B, LOC105611231, ST7L, LOC114116893, LOC132657507, LOC132658352, LOC114115018* |
| ADG | Darkorange | *LOC114108643* |
| ADG | Lightgreen | *NOX4, DSCAML1, LOC132658435, LOC101105064, LOC121817511, URGCP, VSTM2B, NLE1, CFAP77, SERTAD3, EXOSC6, MRPS2, LOC114110907, LOC121816486, TSNARE1, LOC105607754, POLR1E, LOC105604801, LOC105611720, EIPR1, LOC106991840, INTU, SIVA1, LOC114116073, BRINP1, VWA5A, LOC114108720, LSM2, KIF16B, MIOX, ARTN, ZNF304, LIMS2, LOC101106416, LOC121817642, ZNF501, LOC121817090, LOC101105418, TMEM203, SRM* |
| RFI | Lightcyan | *ABCA3, ACER2* |
| RFI | Cyan | *COL5A1, RNF212, LOC114117316, SECISBP2L, LOC121819003, LOC121818811, LOC132660015* |
| RFI | Lightgreen | *NOX4, DSCAML1, LOC132658435, LOC101105064, LOC121817511, URGCP, VSTM2B, NLE1, CFAP77, SERTAD3, EXOSC6, MRPS2, LOC114110907, LOC121816486, TSNARE1, LOC105607754, POLR1E, LOC105604801, LOC105611720, EIPR1, LOC106991840, INTU, SIVA1, LOC114116073, BRINP1, VWA5A, LOC114108720, LSM2, KIF16B, MIOX, ARTN, ZNF304, LIMS2, LOC101106416, LOC121817642, ZNF501, LOC121817090, LOC101105418, TMEM203, SRM* |
| RFI | Magenta | *LTBP4, APEX1, ZNF772, MESP2, CDH17, LOC105605854, LOC121817131, LOC132660240, TFR2, PIK3C2B* |

DGE; Differential gene expression, WGCNA; weighted gene co-expression network analysis, cis-eQTL; cis-expression quantitative trait loci, DMI; dry matter intake, ADG; average daily live weight gain, RFI; residual feed intake.

**Supplementary Figures**

Supplementary Figure S1: Principal component analysis (PCA) plot of the normalized RNA-Seq data showing no clear samples separation by varying levels of microalgae oil. The group here corresponds to the different levels of microalgae oil fed to the lambs (Control;0g/kg dry matter (DM), Low: 5.4g/Kg DM, Medium: 10.8g/Kg DM, High 16.2g/Kg DM). PC1(11%) and PC2 (9%) represents the first and second principal components.

Supplementary Figure S2: Effect of feed efficiency (FE) traits in response to varying levels of microalgae oil supplementation. Box plots show the distribution of DMI_absolute_ (A), DMI_adjusted_ (B), ADG (C), and RFI (D) across the four dietary treatment groups: Control (blue), Low (yellow), Medium (grey), and High (red) microalgae oil inclusion to replace soya oil. Each box represents the interquartile range, with the horizontal line indicating the median and individual points representing sample-level values. An ANOVA test revealed no statistically significant differences among groups, suggesting that increasing levels of microalgae oil did not significantly change FE under the conditions tested.

Supplementary Figure S3: Venn diagram showing distribution of DEGs across the studied FE traits. No overlap was observed between DEGs associated with the analysed four FE traits, indicating that each trait is characterized by distinct transcriptional signatures.

Supplementary Figure S4: Diagram representing the hierarchical clustering tree of the modules identified. The x-axis represents genes and co-expression distance is represented in the y-axis.
